# Advancing drug safety and mitigating health concerns: High-resolution mass spectrometry in the levothyroxine case study

**DOI:** 10.1016/j.jpha.2024.100970

**Published:** 2024-03-28

**Authors:** Hana Chmelařová, Maria Carmen Catapano, Jean-Christophe Garrigues, František Švec, Lucie Nováková

**Affiliations:** a Department of Analytical Chemistry, Faculty of Pharmacy in Hradec Králové, Charles University, Akademika Heyrovského 1203, 500 05, Hradec Králové, Czech Republic; b Laboratoire SOFTMAT (IMRCP), Université de Toulouse, CNRS UMR 5623, Université Paul Sabatier, 118 route de Narbonne, 31062 Toulouse Cedex 9, France

**Keywords:** Levothyroxine, Excipients, Impurities, UHPLC-DIA-HRMS, Drug safety

## Abstract

Levothyroxine is a drug with a narrow therapeutic index. Changing the drug formulation composition or switching between pharmaceutical brands can alter the bioavailability, which can result in major health problems. However, the increased adverse drug reactions have not been fully explained scientifically yet and a thorough investigation of the formulations is needed.

In this study, we used a non-targeted analytical approach to examine the various levothyroxine formulations in detail and to reveal possible chemical changes. Ultra-high-performance liquid chromatography coupled with a data-independent acquisition high-resolution mass spectrometry (UHPLC-DIA-HRMS) was employed.

UHPLC-DIA-HRMS allowed not only the detection of levothyroxine degradation products, but also the presence of non-expected components in the formulations. Among these, we identified compounds resulting from reactions between mannitol and other excipients, such as citric acid, stearate, and palmitate, or from reactions between an excipient and an active pharmaceutical ingredient, such as levothyroxine-lactose adduct. In addition to these compounds, undeclared phospholipids were also found in three formulations. This non-targeted approach is not common in pharmaceutical quality control analysis. Revealing the presence of unexpected compounds in drug formulations proved that the current control mechanisms do not have to cover the full complexity of pharmaceutical formulations necessarily.

## Highlights

• UHPLC-DIA-HRMS was used for detailed examination of levothyroxine formulations.
• New reaction products between excipients in the formulations were identified.
• Previously unreported phospholipids were discovered in several samples.
• The revealed insights hold the potential to enhance drug quality and safety control.

## Graphical abstract

**Figure:**
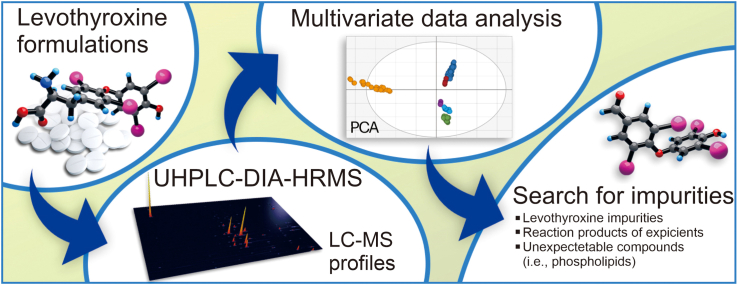

## 1 Introduction

Drug safety is an important issue in medical practice. Therefore, it is thoroughly monitored by the European Medicines Agency (EMA) in the EU and by the Food and Drug Administration (FDA) in the USA. Quality standards for pharmaceutical substances are covered by the European Pharmacopoeia (Ph. Eur.) or US Pharmacopoeia (USP), which also include validated analytical methods for testing pharmaceutical substances. The content and qualification of impurities in new drug substances are addressed by the International Council for Harmonization of Technical Requirements for Pharmaceuticals for Human Use (ICH) guidelines [1]. Despite extensive legislative measures, adverse drug reactions (ADRs) continue to be reported. Levothyroxine is one of the problematic drugs which stands behind several health crises. Levothyroxine is a synthetic hormone, widely prescribed for hypothyroidism treatment. This active pharmaceutical ingredient (API) has a narrow therapeutic index, meaning that a small change in its concentration can significantly affect therapeutic activity. In addition to its potential instability, it is also problematic in terms of bioequivalence when switched to a different formulation [2].

**Fig. 1:**
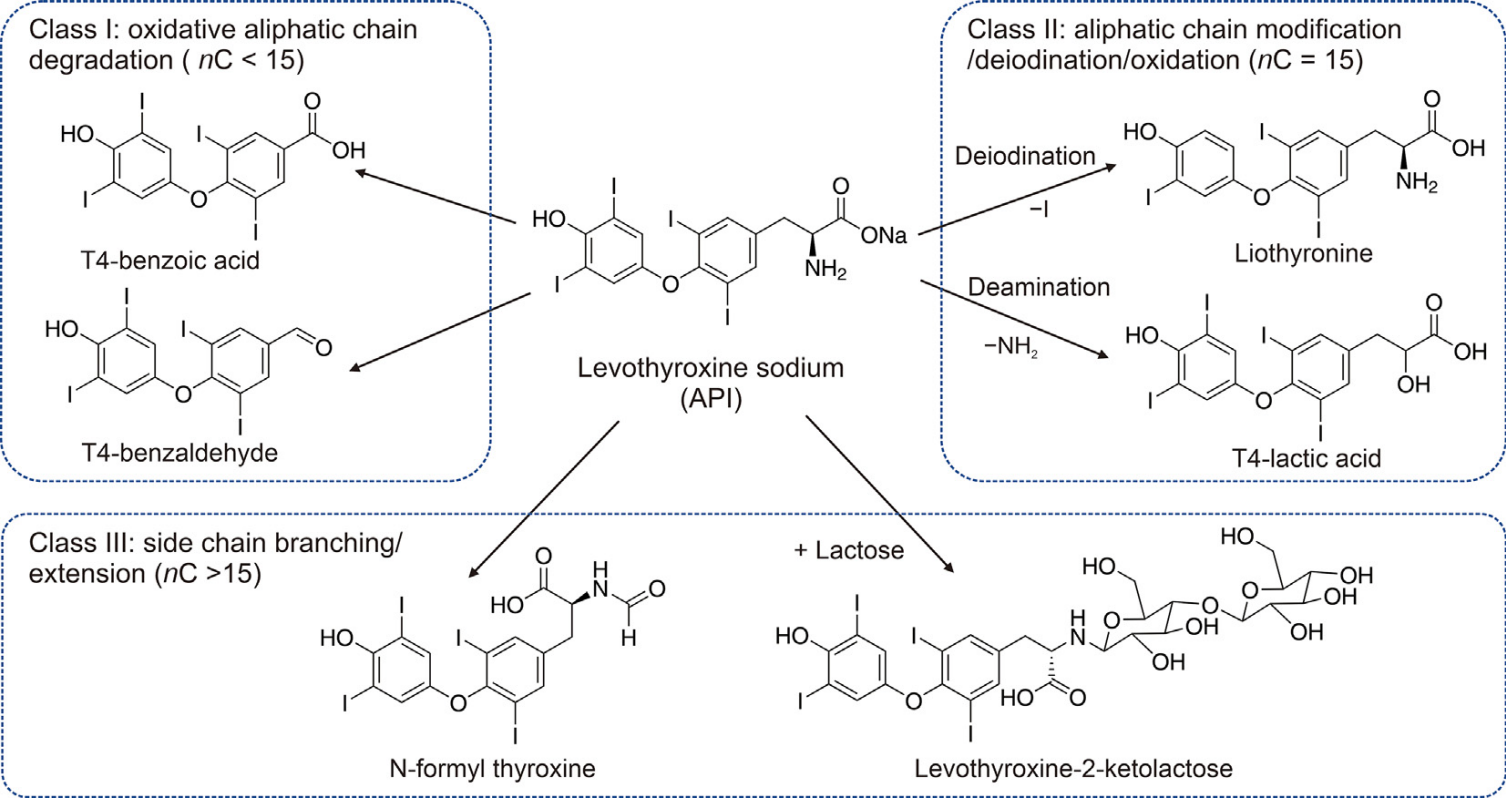
Structure of levothyroxine sodium and examples of its degradation products, which were classified according to Neu et al. [6]. T4: tetraiodothyro-in the name of the structure.

The stability of levothyroxine is an important issue, as it is sensitive to several factors such as light, oxygen, humidity, and pH [[2], [3], [4], [5]]. Degradation of levothyroxine can occur via several pathways, most notably deiodination, oxidation, deamination, decarboxylation, side-chain branching, and dimerization [4,[6], [7], [8]]. Neu et al. [6] have established a classification system of impurities, including five classes according to their structural diversity. The structure of levothyroxine sodium and examples of possible degradation products belonging to the first three classes are shown in Fig. 1, as they are important for this study. A more detailed description of the mechanism of levothyroxine degradation, including a radical-driven reaction scheme, can be found in the work of Neu et al. [9].

The stability and bioavailability of the API may also be affected by excipients. Levothyroxine is a low-dose API where formulations consist predominantly of excipients. For example, a 100 mg tablet with a 100 μg dose of levothyroxine contains 99.9% of excipients [[2], [3], [4],^10^,^11^]. Therefore, great attention must be paid to bioequivalence when reformulating or switching from one brand of levothyroxine to another [10,12]. The effect of excipients on the stability of levothyroxine was evaluated in several systematic studies. Critical stability factors involve microenvironmental acidity and hygroscopicity [[2], [3], [4], [5],^11^,[13], [14], [15]]. Organic acids were found to induce pronounced salt disproportionation of levothyroxine sodium to the free acid form which has a 100-fold lower solubility. Therefore, the bioavailability can be significantly affected [2,11]. Moreover, excipients with possible reactivity with the API are undesirable. Levothyroxine contains a primary amino group that tends to react with reducing sugars via the Maillard reaction. Hence, lactose is an example of an inappropriate excipient [2,16]. Surprisingly, it is still used in some levothyroxine formulations [2].

Because of the potential instability and the risk of decreased potency, regulatory agencies are increasingly adopting more stringent potency specifications for levothyroxine to reduce potential fluctuations in drug concentrations. In 2007, FDA revised the required potency range of levothyroxine during its shelf life from 90%–110% to 95%–105% [2,17,18]. The same stricter specification was adopted in France by the Agence Nationale de Sécurité du Médicament et des Produits de Santé (ANSM) in 2012 [19,20]. To improve levothyroxine stability, the main excipient, lactose, was replaced by mannitol and citric acid in the dominant marketed product, to prevent the Maillard reaction between lactose and levothyroxine [12,[19], [20], [21]]. However, despite the conducted bioequivalence study [22], introducing the new formulation resulted in a significant health crisis. After the substitution and one year of marketing of the new formulation, 1.43% of patients reported ADRs to the French network of pharmacovigilance centers [21]. The same phenomenon was observed in New Zealand and Denmark in 2007 and 2009, respectively [12,23]. Also in these cases, the composition of the levothyroxine drug product was reformulated to improve the stability by replacing lactose with other excipients [23]. The reported ADRs were in a similar range to those in France, at 1%–2% of patients [10,12]. In response to these crises, the European Thyroid Association (ETA) and the Thyroid Federation International (TFI) presented a joint statement and recommendations for switching of levothyroxine formulations in 2018 [12]. Subsequently, when the recommendations of ETA and TFI were already being followed, this crisis was not repeated in other European countries [24]. However, there is no clear scientific evidence why the change in excipient composition can lead to increased ADRs. Therefore, a detailed examination of the composition of levothyroxine formulations is needed.

According to the Ph. Eur., high-performance liquid chromatography with ultraviolet detection (HPLC-UV) at 225 nm [25] is the standardized analytical method for the control of levothyroxine. However, the assay for associated impurities is defined only for levothyroxine as a pure substance. Unlike a regular UV detector, a mass spectrometer (MS) offers better possibilities to elucidate the structure of potential impurities [26]. In LC-MS data, other components without a chromophore, e.g., excipients and their possible impurities and reaction products, can be visible. Several studies have used the LC-MS technique to elucidate the structures of levothyroxine impurities [[6], [7], [8], [9]]. In these studies, high-resolution tandem mass spectrometry (HRMS/MS) using a linear ion trap Orbitrap mass spectrometer was used for a comprehensive structural elucidation of the levothyroxine impurity pattern. However, they were focused on the degradation products of pure levothyroxine, under different conditions, such as batch-to-batch variability [6], aging [8], or thermal stressing [7,9]. Another LC-MS study was focused on the determination of impurities in a levothyroxine oral solution [27]. The stability of levothyroxine in the presence of various excipients and pH modifiers has been investigated in several studies [[3], [4], [5],^11^]. However, artificially prepared binary mixtures were usually tested [4,5,11]. To the best of our knowledge, no published study has addressed the impurities of real marketed products that have been aged under common storage conditions.

The aim of this study was a holistic investigation of levothyroxine drug products using a metabolomic approach employing ultra-high-performance liquid chromatography coupled with a data-independent acquisition high-resolution mass spectrometry (UHPLC-DIA-HRMS). UHPLC-HRMS provides an accurate mass-to-charge ratio (*m/z*) and relative abundance for all the detected features. DIA used in this study includes all-ion fragmentation (MS^E^), which consists of sequential MS and MS/MS scans to obtain all precursor and fragment ions, respectively [[28], [29], [30]]. The analysis was focused on: (i) non-targeted screening of all the detectable compounds to identify possible changes and interactions of API and excipients in the levothyroxine tablets during the storage; (ii) semi-targeted analysis focused on the levothyroxine degradation products. The UHPLC-DIA-HRMS technique combined with multivariate data analysis was successfully applied for both purposes.

## 2 Experimental

### 2.1 Chemicals

Water (Optima LC-MS) and methanol (Optima LC/MS) were obtained from Fisher (FisherScientific, Loughborough, UK), LC-MS grade acetonitrile (ACN) and LC-MS grade formic acid (FA) were purchased from Biosolve (Valkenswaard, The Netherlands). Dimethyl sulfoxide anhydrous (≥99.9%) (DMSO), d-lactose monohydrate (≥99.9%), and leucine-enkephalin acetate salt hydrate (97%) were purchased from Sigma-Aldrich (Darmstadt, Germany), d-mannitol p.a. was purchased from Lach-Ner (Neratovice, Czech Republic). The internal standard, ^13^C_6_ levothyroxine, was purchased from TRC Canada (Toronto, Canada). Sodium hydroxide for the preparation of the sodium formate calibration solution was purchased from Penta (Prague, Czech Republic).

### 2.2 Analyzed samples

A total of 45 batches of levothyroxine commercial samples were analyzed, including 6 different formulations from 5 various manufacturers: Biogaran, Genevrier, Merck, Sanofi, and Uni-Pharma. For one of the manufacturers (M), two different formulations were analyzed. According to their dominant excipient, they are designated as M1L and M1M for lactose and mannitol, respectively. A summary of the sample composition in terms of excipients, based on the manufacturer's information in the package leaflet, is shown in Table 1. The samples were stored in the dark at laboratory temperature (20 °C) in the original blister packs, i.e., without access to air and light. The samples were kindly provided by the French Association of Thyroid Diseases (AFMT) from their archive. Due to the limited availability of some formulations (especially M2) and limited expiration time, some of them were already expired at the time of final analysis. However, the data from the “aged” samples are also valuable for the study.

**Table 1:** A summary of excipient composition in the measured samples.

Manufacturer (number of samples)	Low-molecular filler	Starch	Cellulose and its derivatives	Glidant	pH modifier	Others
M1L (9)	lactose monohydrate	corn starch	sodium croscarmellose	magnesium stearate		gelatin
M1M (20)	mannitol	corn starch	sodium croscarmellose	magnesium stearate	anhydrous citric acid	gelatin
M2 (2)	mannitol		microcrystalline cellulose, hypromellose	magnesium stearate		
M3 (4)		corn starch, pregelatinized corn starch	microcrystalline cellulose	colloidal silica	sodium carbonate anhydrous	sodium thiosulphate, hydrogenated castor oil
M4 (3)			powdered cellulose, sodium croscarmellose, microcrystalline cellulose	magnesium stearate, colloidal silica		
M5 (7)	glycerol					gelatin (capsule), purified water

### 2.3 Internal standard solution

The internal standard (IS) stock solution was prepared by dissolving stable isotopically labeled ^13^C_6_ levothyroxine in MeOH to obtain a solution at a concentration of 1 mg/mL. This solution was then diluted in an extraction solvent (DMSO or 50% MeOH in water) to the concentration of 2.2 μg/mL. This working solution was then used to dilute the sample extracts, as described in the following section.

### 2.4 Sample preparation

The drug formulations including tablets and capsules were subjected to two types of extraction with solvents of different polarities: A) DMSO and B) 50% MeOH in water.

One tablet was dissolved in solvent (DMSO or 50% MeOH) in the ratio of 1 mL of solvent per 100 mg of tablet, supported by vortex (10 s) and ultrasonic bath (10 min), repeated four times. Gelatin capsules were cut in half before extraction. After centrifugation at 5,000 rpm (2,370 *g*) for 15 min, 100 μL supernatant was transferred into amber vials and diluted 10 times with the same solvent (DMSO or 50% MeOH) to which isotopically labeled IS was added. The final concentration of IS in the vials was 2 μg/mL.

Samples were prepared in a random order in two batches. The extracts were injected into the UHPLC-HRMS system in a randomized order in duplicates. After every second sample, a quality control (QC) sample was injected to ensure the analytical procedure was carried out properly. The QC sample was prepared by pooling the aliquots of the final extracts. Blank extracts were processed using the same protocol as the samples, but without drug formulation, and were injected after every fifth sample.

### 2.5 UHPLC-DIA-HRMS analysis

UHPLC-DIA-HRMS analysis was carried out using an Acquity I-class UHPLC system coupled with a SYNAPT G2-Si high-resolution tandem mass spectrometer with quadrupole-time-of-flight (Q-TOF) mass analyzer (Waters, Manchester, UK). The column and gradient conditions were selected to achieve the best possible separation of polar compounds under the reversed-phase conditions. Separation was carried out on an Acquity UPLC HSS T3 column (150 mm × 2.1 mm, 1.8 μm particle size; Waters ) at 40 °C. The mobile phase consisted of A) 0.1% formic acid in water and B) 0.1% formic acid in acetonitrile; the gradient elution profile was as follows: 0–1 min: 0% B; 1–6 min: 0–100% B; 6–10 min: 100% B; 10–13 min: 0% B, at a flow rate 0.5 mL/min. The injection volume was 1 μL. The autosampler temperature was set to 5 °C for MeOH extracts and 20 °C for DMSO extracts due to the high freezing point of DMSO (18.5 °C).

The MS instrument was calibrated over the mass range of *m/z* 50–1200 using 0.5 mmol/L sodium formate solution. A leucine-enkephalin solution (0.2 ng/μL) was used as a lock mass calibrant to correct for small mass drifts and was continuously introduced into the mass spectrometer via the LockSpray™ dual electrospray at a flow rate of 10 μL/min. MS spectra were recorded in the *m/z* range 50–1200, using continuum data acquisition in resolution mode with a mass resolving power of approximately 20,000 full width at half maximum (FWHM). The instrument was operated in both negative and positive electrospray ionization (ESI) modes. The ESI^–^ parameters were set as follows: capillary voltage: –1 kV; cone voltage: –40 V; source temperature: 120 °C; desolvation gas temperature: 600 °C. The desolvation and cone gas (N_2_) flow rates were set to 1,000 and 50 L/h, respectively. The ESI^+^ parameters were kept the same as in the case for ESI^–^, except for the capillary and cone voltages, which were +1 kV and +40 V, respectively. Both MS and MS/MS spectra were acquired in a data-independent acquisition called MS^E^. Full MS spectra were acquired at the low collision energy of 4 eV, while fragmentation was carried out at the ramped collision energy from 30 to 50 eV.

### 2.6 Data processing

Data were acquired by the MassLynx version 4.2 software (Waters) and processed using the UNIFI® software platform (Waters). In the first step, peaks from the acquired continuum data were detected using a 3D peak detection algorithm. The intensity threshold was set to 300 and 100 counts for MS and MS/MS acquisition modes, respectively. In the next step, the detected features defined by retention time, exact mass, and intensity were transferred into a data matrix using the EZinfo extension software (Sartorius, Göttingen, Germany), with individual samples in the rows and aligned features in the columns. Features that were also present in blank extracts were removed as they did not originate from the sample but from the sample preparation procedure.

The resulting data matrix was exported to the SIMCA software (version 16.0.1; Sartorius), which was used for the multivariate data analysis. Before the analysis, the data were scaled to Pareto Variance, where the square root of the standard deviation was used as the scaling factor, which reduced the relative importance of large values but partially preserved the data structure [31]. Principal component analysis (PCA) was employed as an unsupervised modeling approach to visualize the data structure.

### 2.7 Marker identification

The molecular formulas of the selected markers were then estimated using their exact mass, with the mass accuracy window of ±5 ppm, and isotopic pattern. Subsequently, compounds were tentatively identified using a database search such as ChemSpider, PubChem, and Metlin, as well as relevant literature. Identification was supported by the interpretation of MS and MS/MS spectra. The expected compounds were added into the UNIFI® library as molfiles. In case of a match with the precursor mass in the full MS spectrum, automated in-silico fragmentation was carried out to confirm the structure. At least two diagnostic fragments were required for tentative identification of the compound. The exceptions were mannitol palmitate and stearate, which provided only one specific fragment of respective fatty acyl.

## 3 Results and discussion

### 3.1 Optimization of extraction and chromatographic conditions

As a first step, an extraction procedure was optimized to find a suitable solvent to dissolve the samples. Acetonitrile was the first choice because of its universal extraction properties. However, it was found to be unsuitable due to the precipitation of the samples. Significantly better behavior was observed with DMSO. This polar solvent, which is organosulfuric and aprotic, allowed the dissolution of both polar and apolar compounds and was compatible with both the solid tablets and gelatin capsules. However, after the data review, DMSO was found to be incompatible with the API as it was unstable in DMSO extracts (see Section 3.3 for details). Among the other solvents tested, 50% MeOH in water and 100% MeOH, the 50% MeOH was selected as the best option for the formulation dissolving and extracting levothyroxine. Finally, two types of extraction were carried out: i) with DMSO to extract a wide range of impurity polarity and ii) with 50% MeOH in water, which showed no recognized reactivity with the API in an extract but showed lower efficiency for nonpolar impurities. The chromatographic records of DMSO and 50% MeOH extracts are compared in Supplementary material in Fig. S1.

Chromatographic conditions were optimized to achieve the separation of polar components, such as mannitol and citric acid. This separation could not be achieved with the Acquity BEH Shield RP18 column, which was used as the first choice for method development, even when we used a gradient starting with 100% aqueous mobile phase and tested a longer column (100 mm instead of 50 mm). The Acquity HSS T3 column was selected because it is a reversed-phased column designed for improved separation of polar compounds and is suitable for apolar analytes. This column has a lower ligand density allowing secondary interactions of polar compounds with residual silanols. It also enables starting with a 100% aqueous mobile phase, further improving the separation of highly polar compounds. In addition, the longer column (150 mm) provided a higher number of theoretical plates, which further improved separation. For the comparison of the chromatograms obtained from Acquity BEH Shield RP18 (50 mm in length) and Acquity HSS T3 (150 mm in length), please see Fig. S2.

### 3.2 Non-targeted screening

**Fig. 2:**
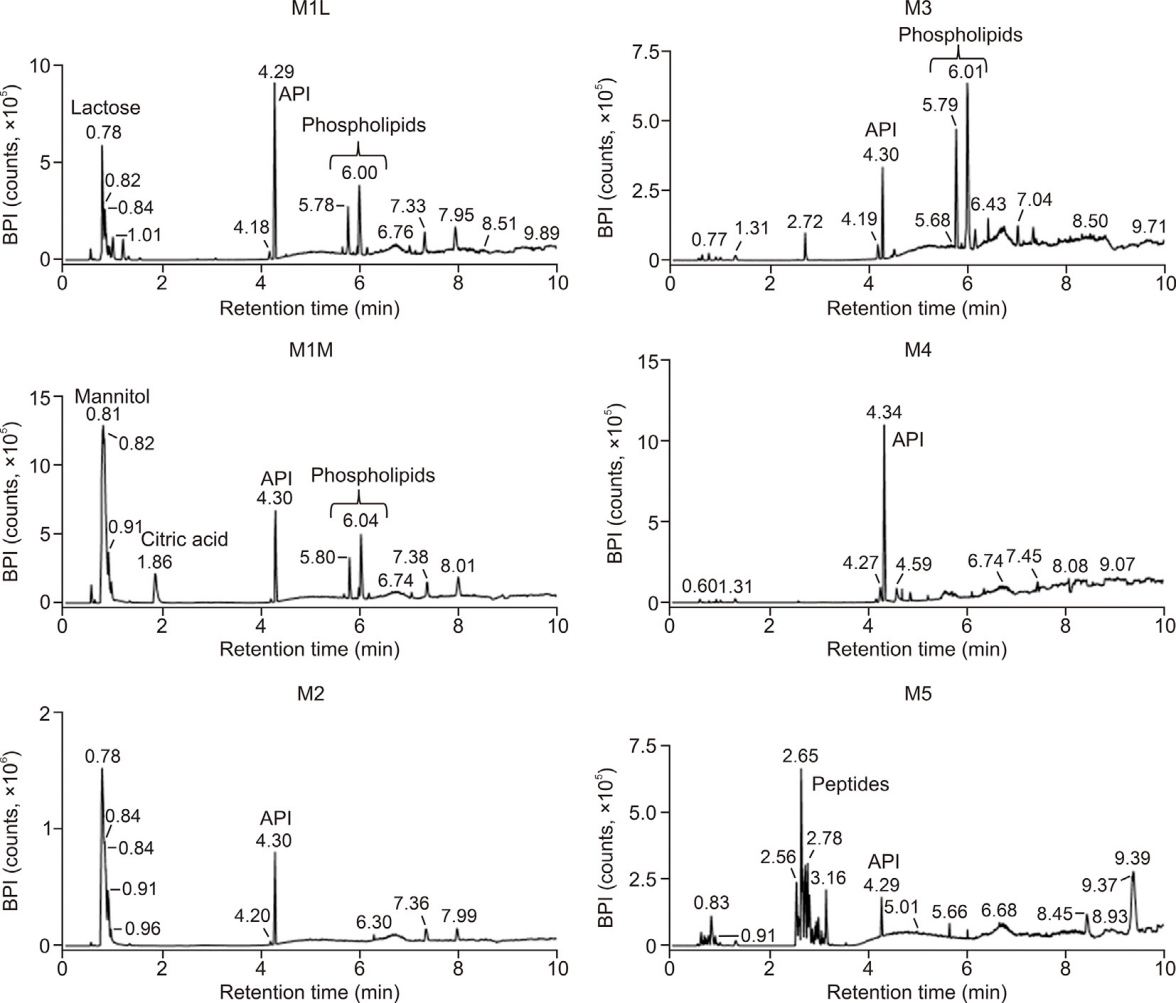
Examples of base peak intensity (BPI) chromatograms for selected levothyroxine formulations of the individual manufacturers.

In this study, we used a comprehensive, metabolomics-like approach to capture as much of the chemical complexity of the formulations as possible. The UHPLC-DIA-HRMS method enabled time-efficient detection of all the small ionizable compounds and simultaneously provided the high-resolution MS and MS/MS spectra. DIA generates MS/MS spectra for all precursor ions, therefore, it is capable of detecting and identifying more metabolites that are at lower concentrations. Thus, in comparison to data-dependent acquisition (DDA), DIA offers greater sensitivity with a minimized risk of data loss [[28], [29], [30]]. The obtained comprehensive information facilitates the identification of the detected components, such as impurities in the formulations. Since the composition of each formulation differs, the UHPLC-HRMS chromatograms are also diametrically different. Examples of base peak intensity (BPI) chromatograms for each formulation are shown in Fig. 2. These chromatograms show the dominant components present in the extracts, in accordance with the declared composition. Among them, polar excipients were eluted within the first 2 min. The peak at retention time (RT) of 0.8 min was identified as mannitol with *m/z* = 181.072 in M1M and M2 samples or as lactose with *m/z* = 341.109 in M1L. The peak at RT = 1.9 min in the chromatogram of M1M was identified as citric acid, with *m/z* = 191.020. Levothyroxine (API) was eluted at 4.3 min. Less polar compounds eluted at later retention times. In the case of gelatin capsules (M5), there are several peaks eluted before API at 2.5–3.5 min. These were tentatively identified as peptides derived from gelatin. For the partial identification, the spectra measured in the positive ion mode were especially useful. In the spectra, multiple charged ions, which are typical for peptides, were observed. Moreover, in MS/MS spectra, this assumption was confirmed by the presence of typical mass differences belonging to the mass of amino acid units and also by the presence of immonium ions. In the chromatograms of M4 samples, a low number of features was detected because these samples consist mainly of high molecular weight excipients such as cellulose or croscarmellose, which were not evident in the measured *m/z* range.

In M1 and M3 samples, abundant peaks were observed eluting around the sixth minute. These ions were identified as phospholipids that were not declared in the formulation composition. Their identification and origin are described in detail in Section 3.2.2. The requirement for the impurity profiles in drug products is covered by ICH guideline Q3B (R2). According to the requirements of this guideline, the other peaks revealed by the analytical procedure should be labeled in the chromatograms and their origin should be discussed. However, impurities arising from excipients present in drug products are not covered by this guideline, and therefore, their presence is not regulated.

#### 3.2.1 Multivariate data analysis

**Fig. 3:**
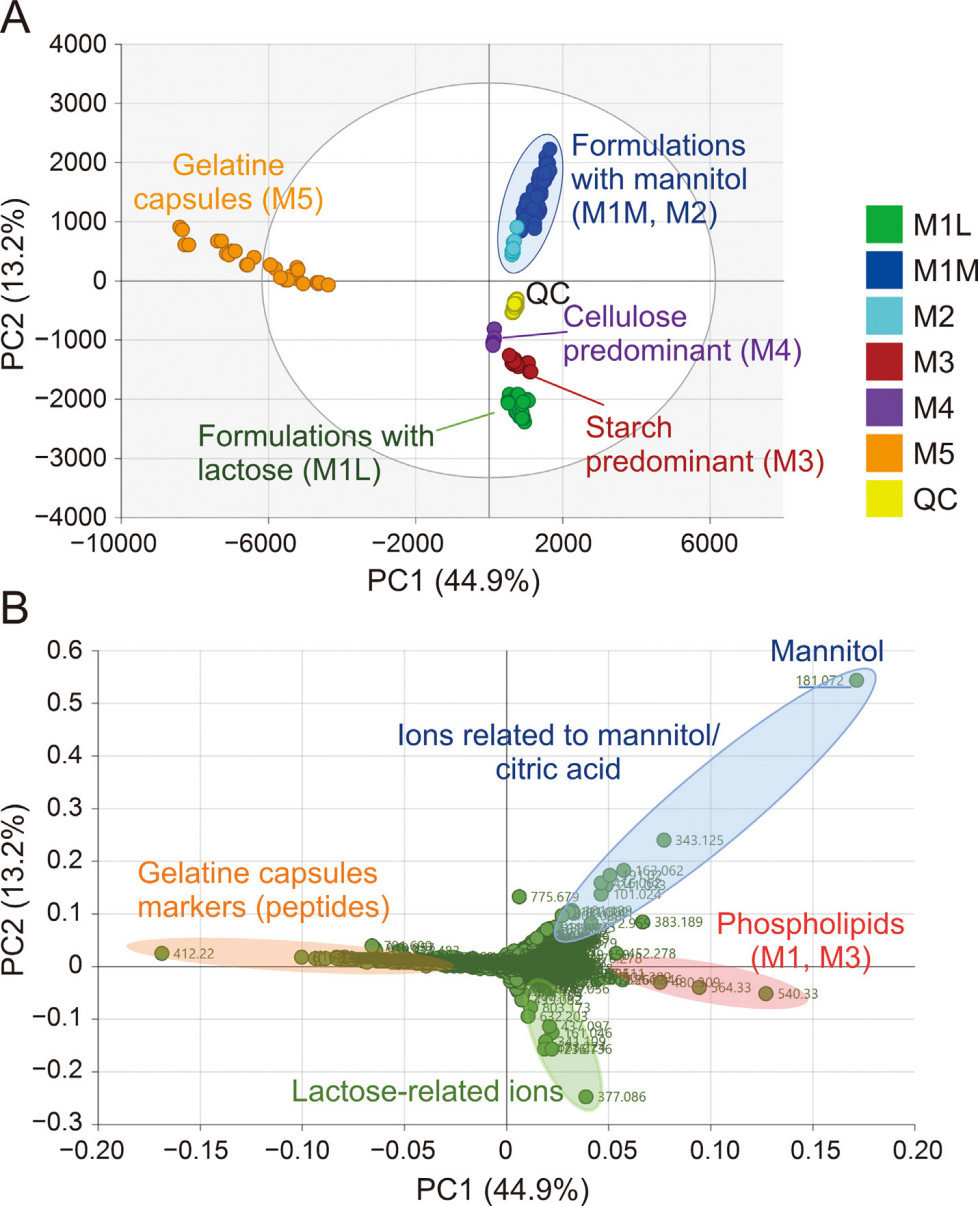
(A) Principal component analysis (PCA) score plot. (B) Loading plot interpreting which ions contribute to the sample positions in the PCA score plot (dimethyl sulfoxide (DMSO) extracts, negative ion mode).

PCA was used as a first step of multivariate data processing to visualize multidimensional data. Expectedly, the clusters were formed according to the composition of the samples. The examples of the PCA score plot and corresponding loading plot for DMSO extracts measured in ESI^–^ mode are shown in Fig. 3.

In this PCA analysis, the samples are represented by the detected features, described by their retention time, *m/z* ratio, and intensity. The formulations of the different manufacturers are differentiated, and, in particular, the formulations with mannitol and with lactose are well separated by the second component (PC2). The first component PC1 separates the gelatin/glycerol formulations from the others. QC points represent quality control samples (pooled sample extracts to confirm that the method is working properly).

Special attention was paid to the samples from manufacturer M1, from whom two different formulations were available. These samples were divided into two groups, M1L and M1M, containing lactose and mannitol as a dominant excipient, respectively. Since the ADRs were observed after reformulation, the differences between these two formulations were examined in detail.

We found that mannitol is probably able to react with stearate to form mannitol stearate ester (*m/z* = 447.3322 min; RT = 7.08 min; C_24_H_47_O_7_) and with citric acid to form mannitol citrate ester (*m/z* = 355.0881 min; RT = 1.98; C_12_H_19_O_12_). In addition, mannitol palmitate ester (*m/z* = 419.3009; RT = 6.56 min; C_22_H_43_O_7_) was tentatively identified. The source of palmitate is also the excipient magnesium stearate, which, according to the pharmacopoeia, is a mixture of stearate and palmitate [32]. These tentatively identified compounds were found in all the samples of M1M. Mannitol stearate and palmitate were also found also in the samples of M2, where their precursors were also included in formulations. A higher intensity was observed in the samples with a longer time after expiration, produced in 2017 and 2018. The opposite trend was observed for citric acid, which was found at significantly higher levels in the samples produced in 2019 and later, in comparison with the samples produced earlier, i.e., longer expired. This may indicate the loss of citric acid during the time due to its reaction with mannitol. It should be noted that these impurities were already found in the samples during earlier experiments, i.e., at a time when they had not yet expired. These trends are shown in Fig. S3 and the detailed mass spectral information including mass errors and fragments is in Tables S1–S3 and Figs. S4–S6.

#### 3.2.2 Phospholipids in levothyroxine formulations

**Fig. 4:**
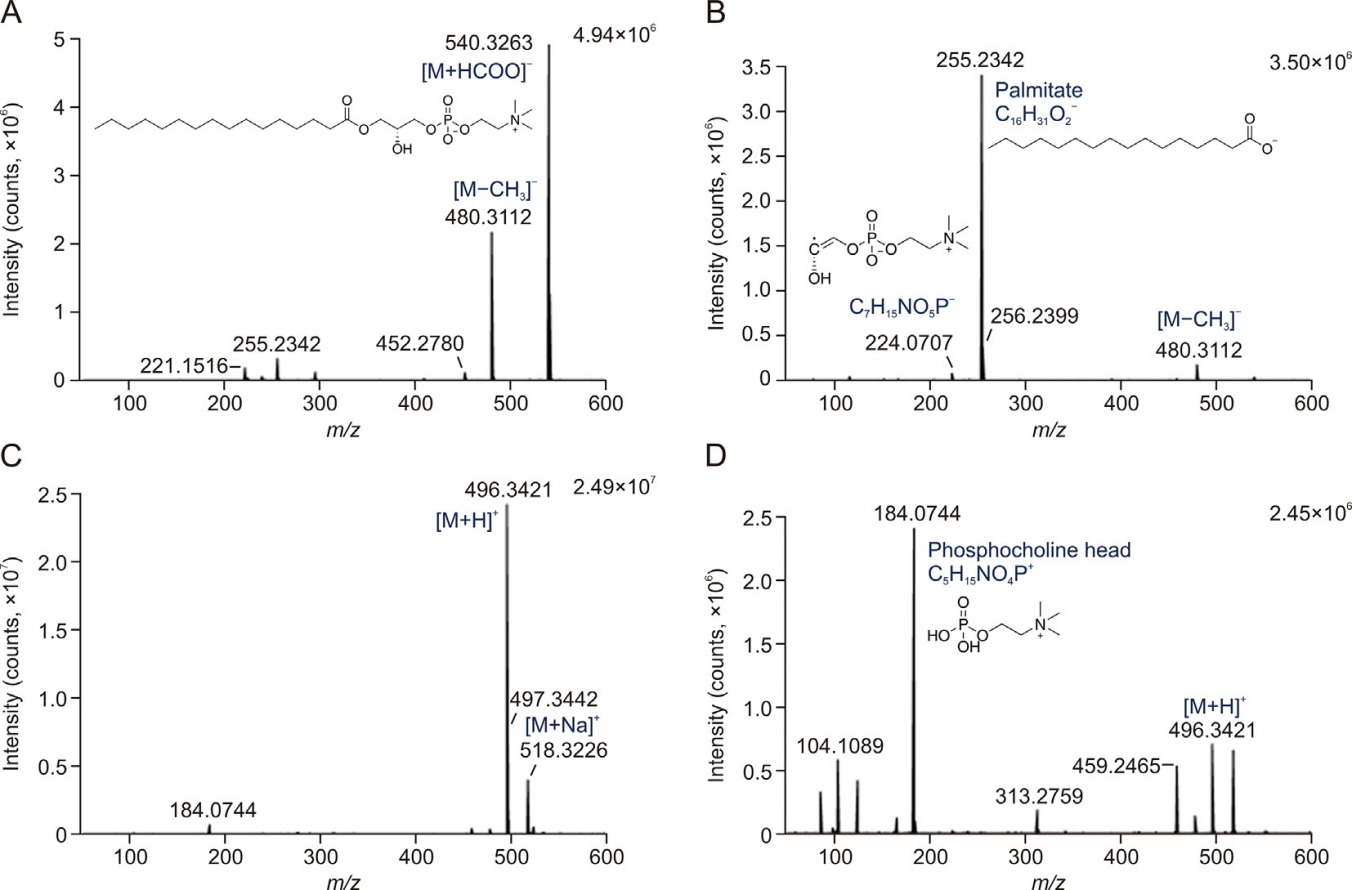
Examples of MS spectra and MS/MS spectra corresponding to lysophosphatidylcholine (LPC) (16:0). (A) electrospray ionization (ESI)^–^, MS (precursor ions), (B) ESI^–^, MS/MS (fragment ions), (C) ESI^+^, MS (precursor ions), and (D) ESI^+^, MS/MS (fragment ions).

Several ions identified as phospholipids were found at high intensity exclusively in the samples from manufacturers M1 and M3, more significant in DMSO extracts, and eluted around the sixth minute. These ions were identified as lysophosphatidylcholines (LPC) and lysophosphatidylethanolamines (LPE), both with fatty acids C16:0, C18:2, and C18:1 in the molecule. LPC were found in negative ionization mode in the ion forms [M+HCOO]^–^ and [M−CH_3_]^–^, while LPE formed a common deprotonated form [M−H]^–^. The formate adducts and demethylated ions were reported as typical for LPC in ESI^–^ [33]. In the positive ion mode, the ions corresponding to the protonated molecule [M + H]^+^ and the sodium adduct [M + Na]^+^ were found. The identification was confirmed by the exact mass (< 5 ppm) and fragmentation spectra in both ionization modes. In ESI^–^, the corresponding fatty acid was a dominant fragment. For LPC, a typical fragment corresponding to the phosphocholine head at *m/z* = 184.074 dominated in ESI^+^. The phospholipid ions were found in two retention times, probably corresponding to two isomers with the acyl moieties at the sn-1 or sn-2 position of the glycerol backbone. An overview of the identified lysophospholipids is given in Table 2, and the interpretation of the mass spectra with identified fragments of LPC (16:0) is shown in Fig. 4. Detailed spectral information of all detected phospholipids, including their fragments, is provided in Tables S4–S9 and Figs. S7–S15.

**Table 2:** Overview of identified phospholipids in M1 and M3 formulations.

Retention time (min)^a^	Negative ion mode		Positive ion mode		Molecular formula	Identification
	Measured *m/z* (mass error)	Ion form	Measured *m/z* (mass error)	Ion form		
5.95; **6.08**	480.3090 (−1.23 ppm)	[M−CH_3_]^–^	496.3405 (1.43 ppm)	[M + H]^+^	C_24_H_50_NO_7_P	LPC (16:0)
	540.3303 (−1.54 ppm)	[M+HCOO]^–^				
5.74; **5.84**	504.3090 (−1.17 ppm)	[M−CH_3_]^–^	520.3398 (1.55 ppm)	[M + H]^+^	C_26_H_50_NO_7_P	LPC (18:2)
	564.3303 (−0.69 ppm)	[M+HCOO]^–^				
6.12; **6.24**	506.3247 (−1.07 ppm)	[M−CH_3_]^–^	522.3554 (0.88 ppm)	[M + H]^+^	C_26_H_52_NO_7_P	LPC (18:1)
	566.3463 (0.00 ppm)	[M+HCOO]^–^				
5.88; **6.00**	452.2780 (−0.62 ppm)	[M−H]^–^	454.2933 (1.03 ppm)	[M + H]^+^	C_21_H_44_NO_7_P	LPE (16:0)
5.78; **5.90**	476.2779 (−0.63 ppm)	[M−H]^–^	478.2934 (1.21 ppm)	[M + H]^+^	C_23_H_44_NO_7_P	LPE (18:2)
6.05; **6.16**	478.2933 (−1.18 ppm)	[M−H]^–^	480.3086 (0.29 ppm)	[M + H]^+^	C_23_H_46_NO_7_P	LPE (18:1)

a Two peaks were found for each detected phospholipid, the dominant one is in bold.

These phospholipids are naturally present in corn endosperm. Therefore, they are most likely originating from corn starch, which is used as an excipient in the evaluated formulations. This assumption is supported by many studies showing that certain lipids, exclusively monoacyl lipids like lysophospholipids, monoacylglycerols, or free fatty acids, form complexes with starch, where the aliphatic chain of the lipid is inserted into the internal cavity of the amylose helix [[34], [35], [36]]. The average signal ratio of the detected LPC C16:0/C18:2/C18:1 was 56/32/12, and LPE C16:0/C18:2/C18:1 was 63/30/7. These ratios were very similar across all the samples. Although the representation of the fatty acids does not match the fatty acids composition of maize, it has been confirmed that LPC (16:0) is trapped in starch granules preferentially [34].

Phospholipids are natural compounds and do not pose any health risk to the drug user. However, their presence can affect the bioavailability of the API due to their amphiphilic properties and ability to form micelles. Thus, they can enhance the dissolution and permeation profile of drugs [[37], [38], [39]]. Moreover, LPCs belong to the class of zwitterionic lipids, and Aleskndrany et al. [40] demonstrated possible interactions between them and levothyroxine, such as hydrophilic, hydrophobic, and van der Waals interactions.

To the best of our knowledge, the presence of polar lipids in levothyroxine formulation or any other starch-based pharmaceutical formulation has never been neither evaluated nor reported. They could not be detected by the official pharmacopeial LC-UV method due to the lack of a chromophore. Also, the requirement for starch in the European pharmacopoeia does not include purity control in terms of the presence of lipids [41]. However, they should be given much attention, especially since levothyroxine is a low-dose API. Hence, the level of phospholipids in the formulation can easily exceed the level of API. For example, if starch contains 0.5% of phospholipids [42] and levothyroxine tablet contains 25 mg of starch [43], the phospholipid content in the tablet is then 125 μg, while the API content is between 25 and 200 μg. Thus, the molar ratio can easily be higher than 1:1. The phospholipid content in starch correlates with the amylose content [36], which can vary from batch to batch. In addition, the presence of phospholipids in starch should be considered when changing the starch content or its form in a drug formulation. For example, the new formulation of levothyroxine introduced in New Zealand in 2007 contained pregelatinized corn starch instead of native corn starch [23], which can change the phospholipid content [44]. Similarly, in France in 2017, the amount of starch was also changed: from 25 mg per tablet used in M1L to 20 mg per tablet used in M1M according to the ANSM analysis report [43].

The same attention should be paid to the amphiphilic impurities mannitol stearate and mannitol palmitate, which are present in higher relative amounts in M1M with an older manufacturing date. They may also interact with API and with other amphiphilic compounds such as phospholipids. To date, no data on these interactions are available in the literature.

### 3.3 Levothyroxine-derived impurities: semi-targeted approach

The chromatographic records were examined with a focus on iodinated compounds, which are considered to be impurities/degradation products of levothyroxine, to investigate the presence of levothyroxine-derived impurities in the measured drug product extracts. Since these impurities represent a minority of the total compounds detected, a semi-targeted approach must have been used. Organic compounds containing iodine have some special mass spectral characteristics that are helpful in their identification by UHPLC-HRMS. They are susceptible to homolytic cleavage due to the relatively weak covalent bond and can therefore be easily detected via their specific fragment ion [I]^–^ at *m/z* = 126.9050 present in MS/MS spectra [45]. In addition, compounds containing iodine show a significant negative mass defect, which is multiplied by the number of iodine atoms bound in the molecule [46]. That means that the value after the decimal point is significantly reduced: each iodine atom gives a mass defect of –x.095; i.e., in the case of four iodine atoms in the molecule, the resulting exact mass is usually xxx.6xx. In the mass spectra of iodine compounds, also a neutral loss of iodine [M–H–I]^–^ is often observed so that the fragment ion has a typical mass difference from the molecular ion [M−H]^–^, whose Δ*m/z* is equal to 126.9045. This examination of the UHPLC-DIA-HRMS data was carried out using the UNIFI elucidation tools “Common fragment search” and “Neutral loss”.

Upon examination of the data obtained from the analysis of DMSO extracts, it was found that levothyroxine is not stable and tends to react with DMSO. In fact, two sulfur-containing compounds were identified in relatively high abundance: the compound eluted at RT = 5.69 min with *m/z* = 819.6515, identified as C_16_H_11_I_4_NO_4_S and the compound eluted at RT = 4.84 min with *m/z* = 869.6519, identified as C_16_H_13_I_4_NO_7_S. These compounds were significantly more abundant in the chromatograms of manufacturers M2, M3, and M4 than in those of M1 and M5. The same reaction products were also found for the internal standard (^13^C_6_-levothyroxine), which confirmed our presumption. All these samples had one common feature in their composition: cellulose as an excipient. Therefore, it can be assumed that the presence of cellulose can somehow accelerate the reaction between levothyroxine and DMSO. Hence, DMSO was evaluated as an inappropriate solvent for the study of levothyroxine degradation impurities. Differences in the stability of levothyroxine in an extract in the presence of different excipients were also reported by Gupta et al. [47], but without specifying the composition of the excipient.

The extraction was then repeated with 50% MeOH. In this case, a better stability of the extracts was confirmed since the reaction during the extraction was avoided. In addition, the temperature of the autosampler could be set to a lower temperature, which can be beneficial for the stability of the extracts. The comparison of extracted ion chromatograms (XIC) in MS/MS with extracted mass 126.905, belonging to [I]^–^ of DMSO and 50% methanolic extracts is provided in Supplementary material in Fig. S16.

**Fig. 5:**
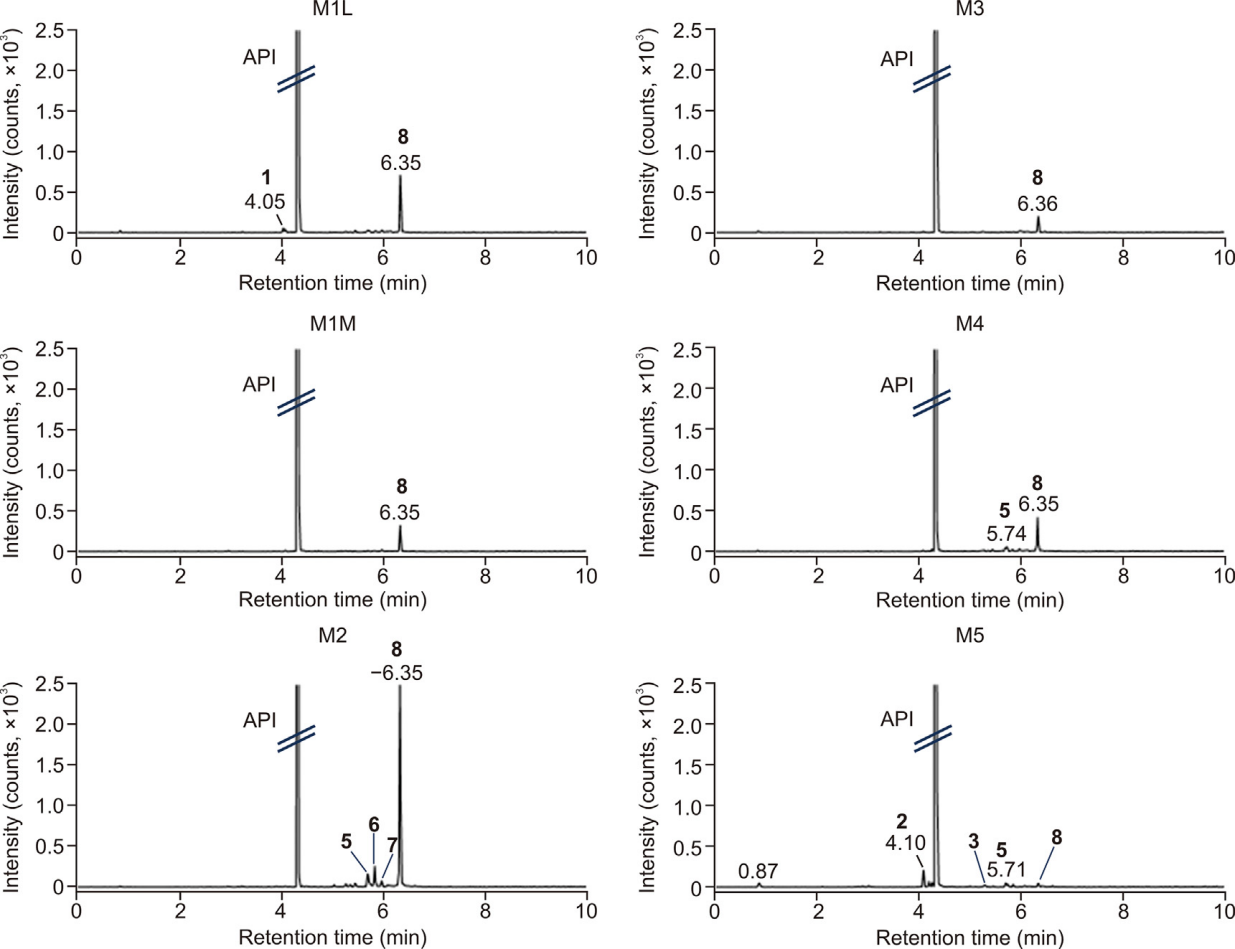
Examples of extracted ion chromatogram (XIC) in electrospray ionization (ESI)^–^ for fragment [I]^–^ at *m/z* = 126.905 for all the formulations. Labeled peaks = dominant detected impurities: 1: levothyroxine-lactose adduct; 2: liothyronine; 5: 2-(4-(4-hydroxy-3,5-diiodophenoxy)-3,5-diiodophenyl)-2-oxoacetamide; 6: 4-(4-hydroxy-3,5-diiodophenoxy)-3,5-diiodo benzoic acid; 8: 4-(4-hydroxy-3,5-diiodophenoxy)-3,5-diiodo benzaldehyde.

The levothyroxine-derived impurities found in 50% MeOH extracts of tablets and capsules from all the manufacturers are compared in Fig. 5.

The tentatively identified impurities together with their structures are listed in Table 3, and their spectra and structure elucidation are shown in Tables S10–S17 and Figs. S17–S27. The mass errors for these impurities were <3 ppm. It should be noted that we detected a significantly lower number of impurities compared to published studies dealing with LC-HRMS/MS elucidation of levothyroxine degradation products [[6], [7], [8], [9]]. These studies dealt with pure synthetic levothyroxine, while in our study, real samples with highly diluted API were analyzed. According to the classification system established by Neu et al. [6], most of the detected impurities can be classified into class I (oxidative side-chain degradation), while Liothyronine can be classified as class II (deiodination), and lactose adduct can be classified as class III. An overview of the relative intensities in relation to the measured intensity of API is given in Table S18. It should be noted that the relative intensities are calculated from the MS responses, which do not reflect the actual concentrations of impurities. It is, therefore, only an approximation of the trends.

**Table 3:** The list of detected levothyroxine-derived impurities.

No.	Retention time (min)	Precursor ion (mass error)	Molecular formula (neutral)	Tentative identification	Estimated structure
1	4.05	[M–H]^–^ 1099.7850 (−0.09 ppm); **[M+H]**^**+**^ 1101.8009 (0.18 ppm)	C_27_H_31_I_4_NO_14_	levothyroxine lactose adduct	
2	4.10	[M–H]^–^ 649.7831 (0.62 ppm); [M+H]^**+**^ 651.7982 (2.30 ppm)	C_15_H_12_I_3_NO_4_	liothyronine (EP impurity A)	
3	5.39	[M–H]^**–**^ 762.6484 (0.92 ppm); [M+H]^+^ n.d.^a^	C_14_H_8_I_4_O_5_	2-hydroxy-2-(4-(4-hydroxy-3,5-diiodophenoxy)-3,5-diiodophenyl) acetic acid	
4	5.56	[M–H]^–^ 745.6690 (0.27 ppm); [M+H]^+^ 747.6845 (1.47 ppm)	C_14_H_9_I_4_NO_3_	2-(4-(4-hydroxy-3,5-diiodophenoxy)-3,5-diiodophenyl) acetamide	
5	5.70	[M–H]^–^ 759.6487 (0.77 ppm); [M+H]^+^ n.d.^a^	C_14_H_7_I_4_NO_4_	2-(4-(4-hydroxy-3,5-diiodophenoxy)-3,5-diiodophenyl)-2-oxoacetamide	
6	5.84	[M–H]^–^ 732.6379 (0.96 ppm); [M+H]^+^ n.d.^a^	C_13_H_6_I_4_O_4_	4-(4-hydroxy-3,5-diiodophenoxy)-3,5-diiodo benzoic acid (EP impurity H)	
7	6.13	[M–H]^–^ 730.6585 (0.75 ppm); [M+H]^+^ n.d.^a^	C_14_H_8_I_4_O_3_	4-(4-hydroxy-3,5-diiodophenoxy)-3,5-diiodo acetaldehyde	
8	6.35	[M–H]^–^ 716.6421 (−0.28 ppm); [M+H]^+^ n.d.^a^	C_13_H_6_I_4_O_3_	4-(4-hydroxy-3,5-diiodophenoxy)-3,5-diiodo benzaldehyde (EP impurity I)	

a n.d. = not detected.

The highest levels of levothyroxine-derived impurities were found in M2 formulations. However, these samples were several years (5 and 7 years) after their expiration date at the time of measurement. On the other hand, contrary to our expectations, there was no correlation between impurity levels and time since the expiration date for the other formulations. M2 samples had disproportionately higher relative levels of the detected levothyroxine-based impurities in comparison to the oldest M1M samples, which were almost 4 years past their expiration date. Additionally, no significant increase in levothyroxine degradation products was observed for the M1M samples. Therefore, API in those samples was stable even long after the expiration date.

The impurity at *m/z* = 716.6421 and RT = 6.12 min had the highest MS response across all the tablets measured but was minor in capsules. It was identified as 4-(4-hydroxy-3,5-diiodophenoxy)-3,5-diiodo benzaldehyde, referred to as “impurity I” in the European Pharmacopoeia (EP) [25]. In the gelatin capsules, liothyronine (triiodothyronine; EP impurity A) was found to be the impurity with the highest relative response compared to the other formulas. Other deiodination products reported in the literature [6,14,26,48], such as diiodothyronine, were not detected in the analyzed samples. The levothyroxine-lactose adduct (*m/z* = 1099.7856 and RT = 4.05 min) was the specific impurity of the lactose-containing formulations.

### 3.4 Possible effects of impurities in the drug formulations

“Why the change in the composition of levothyroxine formulations caused the important ADRs in recent cases?” This was the leading question motivating this study. The UHPLC-DIA-HRMS method enabled the detection of degradation products of levothyroxine. Since the reformulation of levothyroxine products was carried out to increase the stability of levothyroxine, this aspect was very well handled by the manufacturers [22]. Thus, the important ADRs could be explained by the different bioavailability of the API after reformulation.

After a detailed review of the literature and an examination of the extensive UHPLC-DIA-HRMS data, we found several points that could explain the different bioavailability of levothyroxine after reformulation. The bioavailability could be affected by several different reasons:

i) The changed composition of the excipients and its possible effect on the dissolution profile and intestinal absorption.
ii) The presence of citric acid as an acidic pH modifier, which is generally considered to be an inappropriate excipient for levothyroxine because the acidic microenvironment can cause salt disproportionation to the free acid form. The free acid form has significantly lower solubility and the bioavailability may be affected [4,11].
iii) The presence of unexpected compounds with an amphiphilic character that may affect intestinal absorption, especially the lysophospholipids and reaction products of excipients such as mannitol stearate and mannitol palmitate.

Despite the existence of many studies concerning the effect of excipients on levothyroxine stability, there is a lack of studies dealing with the effect of excipients and reactive impurities on levothyroxine bioavailability. In addition, the presence of phospholipids incorporated into tablets together with starch, has never been considered. Therefore, the possible effects of these impurities, which were found in some of the formulations studied, on the bioavailability of levothyroxine should be further investigated.

## 4 Conclusions

This work was designed as an independent study with the aim to comprehensively investigate levothyroxine formulations and thus contribute to their quality and safety in response to previous significant health crises. For this purpose, we developed a new non-targeted approach using UHPLC-DIA-HRMS, an analytical approach not commonly carried out in pharmaceutical QC analysis.

We have demonstrated that the holistic UHPLC-DIA-HRMS approach provides an effective tool for the characterization of pharmaceutical formulations, revealing the presence of new components that were previously overlooked. A comprehensive study of levothyroxine formulations revealed the presence of previously unreported compounds, such as excipient reaction products or undeclared lysophospholipids in starch-containing tablets. These unexpected compounds would not be detected by the standard analytical method LC-UV due to the lack of chromophores. Although the excipients used in new formulations of established drugs met pharmacopeial requirements, the overall context of the drug must be considered. That is particularly important for low-dose narrow therapeutical index drugs where excipients significantly exceed the API, as in the case of levothyroxine.

Impurities in drug products are covered by ICH guidelines, such as ICH Q3A and Q3B, but these focus primarily on the control of impurities in new drug substances and new drug products, respectively. For long-established excipients, such as starch, which are widely used in pharmaceutical formulations, the specific control of impurities is not explicitly covered by these guidelines or pharmacopoeia. The same applies to impurities resulting from reactions between excipients, which are also currently neglected.

In summary, the use of the non-targeted approach helped to reveal the reaction processes taking place in the tablets and also the presence of unexpected compounds. Such findings may be helpful in assessing the bioequivalence of different formulations of levothyroxine, improving the control mechanisms in general, and thus improving the safety of future products.

## CRediT author statement

**Hana Chmelařová:** Data curation, Formal analysis, Investigation, Methodology, Validation, Writing – original draft, Writing – review & editing. **Maria Carmen Catapano:** Data curation, Formal analysis, Investigation, Writing – review & editing. **Jean-Christophe Garrigues:** Conceptualization, Methodology, Writing – original draft, Writing – review & editing. **František Švec:** Conceptualization, Funding acquisition, Writing – review & editing. **Lucie Nováková:** Conceptualization, Data curation, Funding acquisition, Investigation, Methodology, Project administration, Supervision, Writing – original draft, Writing – review & editing.

## Declaration of competing interest

The authors declare that there are no conflicts of interest.
