# Supplementary material for: Advancing drug safety and mitigating health concerns: High-resolution mass spectrometry in the levothyroxine case study
Source: J Pharm Anal. 2024 Mar 28;14(9):100970. doi: 10.1016/j.jpha.2024.100970 (PMC11440252; doi:10.1016/j.jpha.2024.100970)
Supplement: Multimedia component 1 [file mmc1.pdf]

Supplementary information

Advancing drug safety and mitigating health concerns: High-resolution mass spectrometry in the levothyroxine case study

Mass spectral information of the detected impurities found in levothyroxine drug products

A) reaction products of excipients

B) phospholipids

C) Levothyroxine-derived impurities

Part A: optimization of the separation:

- **Figure S1: comparison of separation on BEH Shield RP18 and HSS T3 column**
- **Figure S2: comparison of chromatograms of DMSO and 50% MeOH extracts**

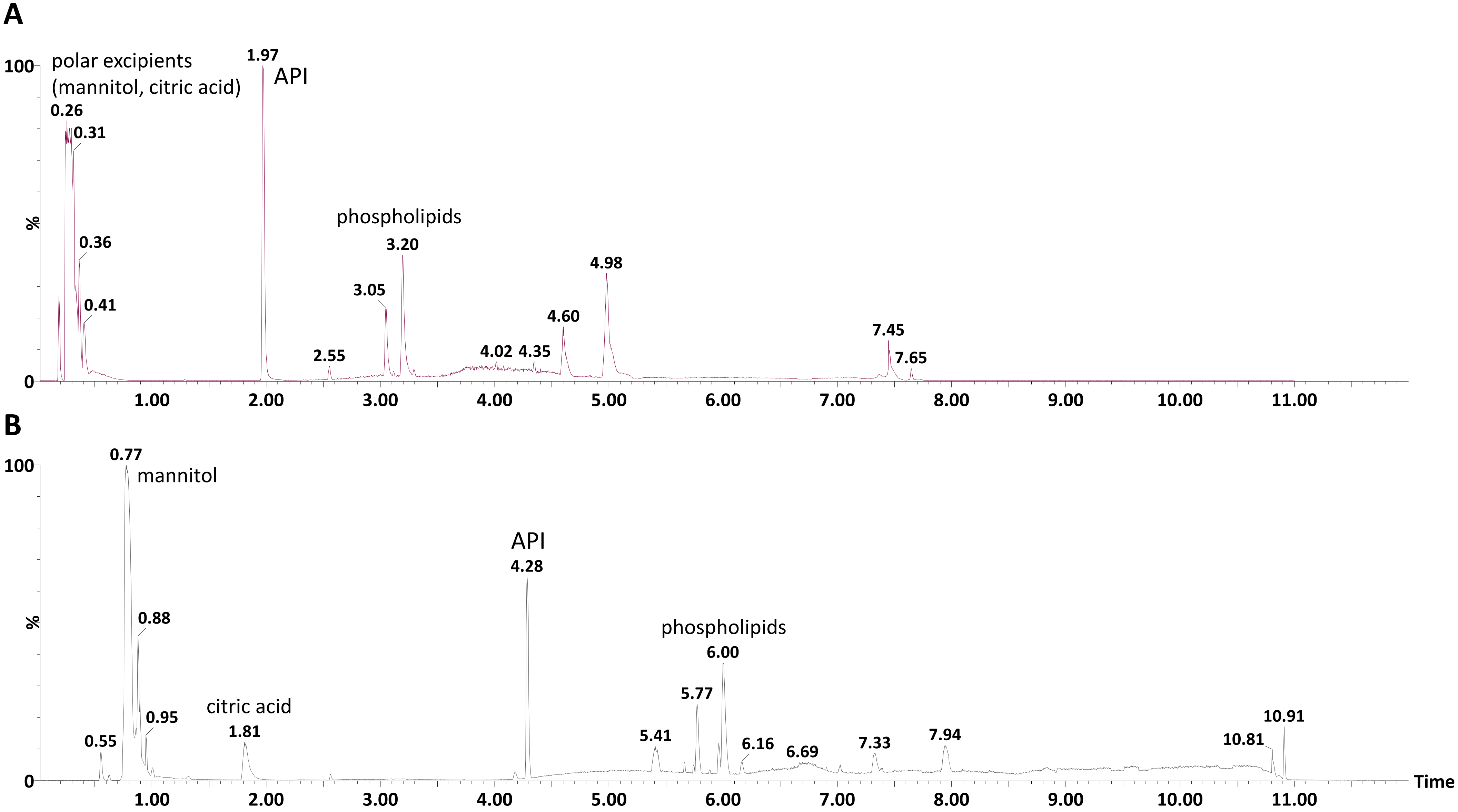

Figure S1: comparison of separation of sample M1M on A) Waters Acquity UPLC BEH Shield RP18 column (2.1 mm x 50 mm; 1.7 µm particle size); B) Waters Acquity UPLC HSS T3 column (2.1 mm x 150 mm; 1.8 µm particle size)

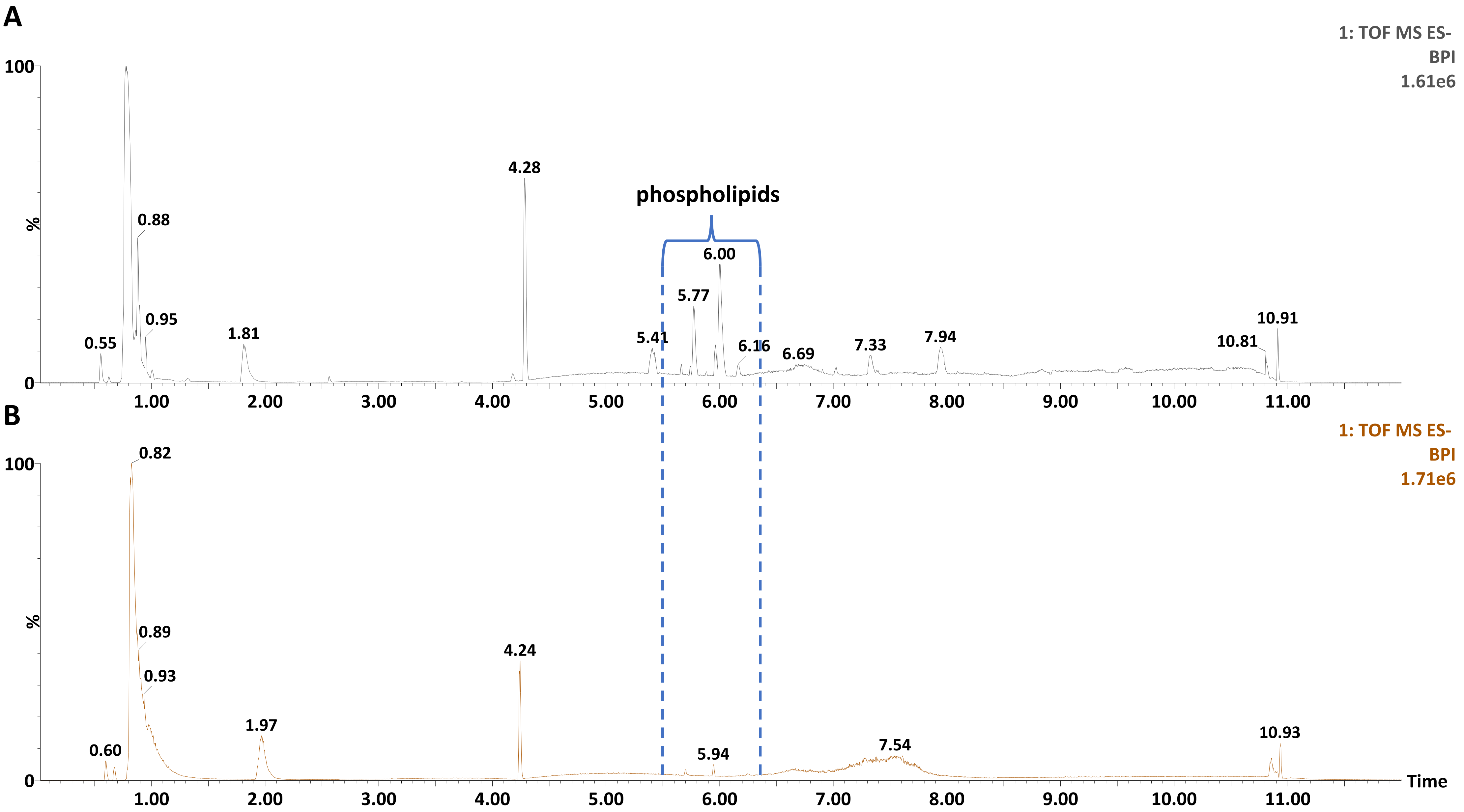

Figure S2: comparison of base peak ion UHPLC-HRMS chromatograms of A) DMSO and B) 50% MeOH extracts of sample M1M

Part A: reaction products of excipients:

- Figure S3: time trends of the reaction products of excipients
- Table S1, Figure S4: mannitol citrate
- Table S2, Figure S5: mannitol stearate
- Table S3, Figure S6: mannitol palmitate

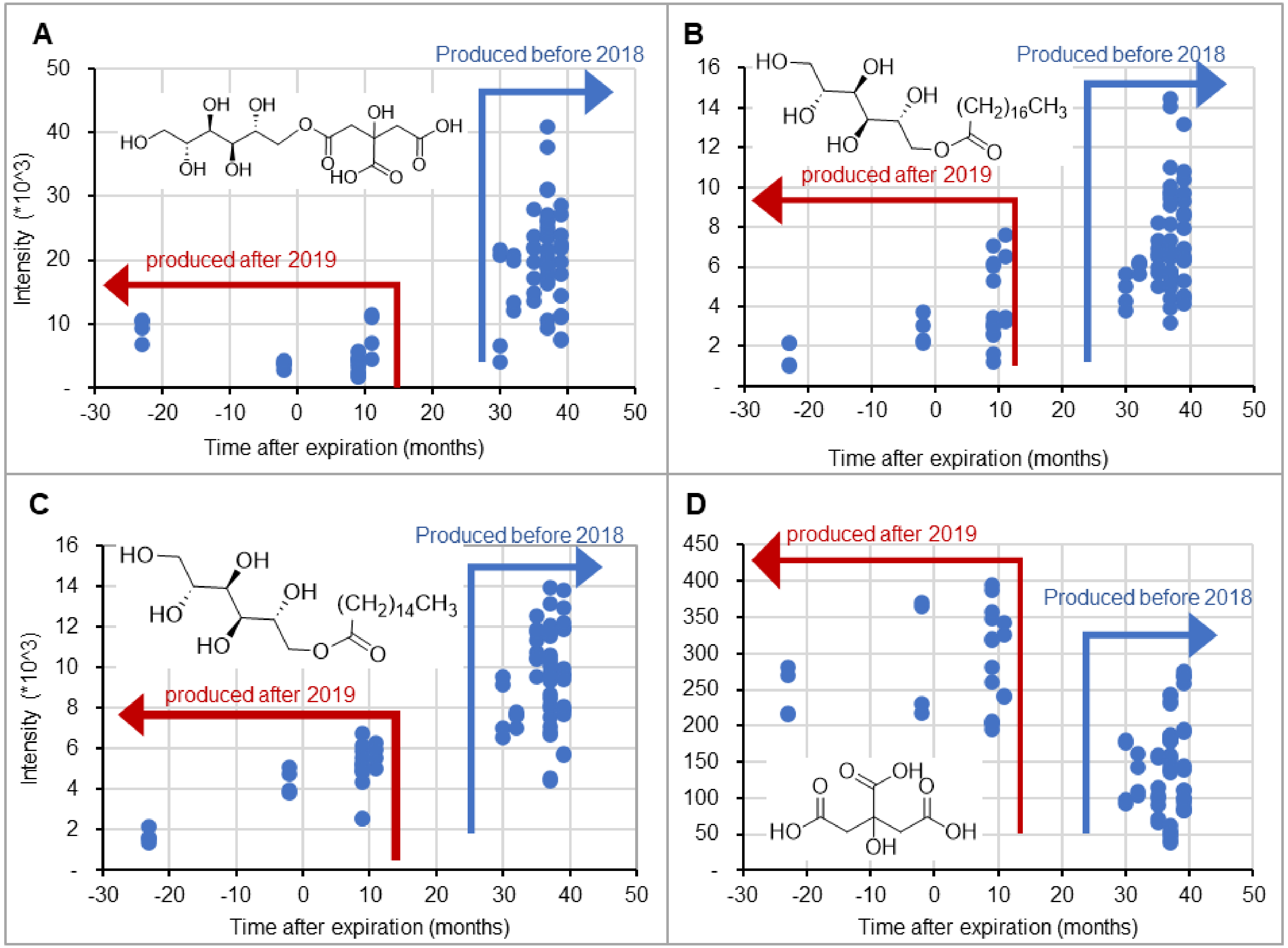

Figure S3: Intensities of reaction products and citric acid found in M1M and their trends as a function of months after expiration at the time of measurement: A) mannitol citrate; B) mannitol stearate; C) mannitol palmitate; D) citric acid. Negative values = measurements were performed before expiration.

Table S1: Mass spectral information of mannitol citrate

**mannitol citrate**			
**molecular formula:** $C_{12}H_{20}O_{12}$	**estimated structure:** 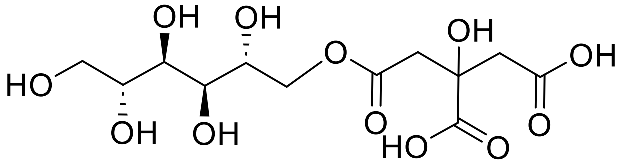		
**retention time (min): 2.17**			
**negative ion mode**		**positive ion mode**	
**parent mass in MS spectra:**	**mass error (Δppm):**	**parent mass in MS spectra:**	**mass error (Δppm):**
**$[M-H]^-$ 355.0881**	-0.15	n.d.	-
**fragment ions in MS/MS spectra:**	**neutral loss:**	**fragment ions in MS/MS spectra:**	**neutral loss:**
191.021 ($C_6H_7O_7$)<br>**111.007 ($C_5H_3O_3$)**	mannitol<br>**mannitol, CO_2, $2H_2O$**	-	-

*bold = the most abundant fragment

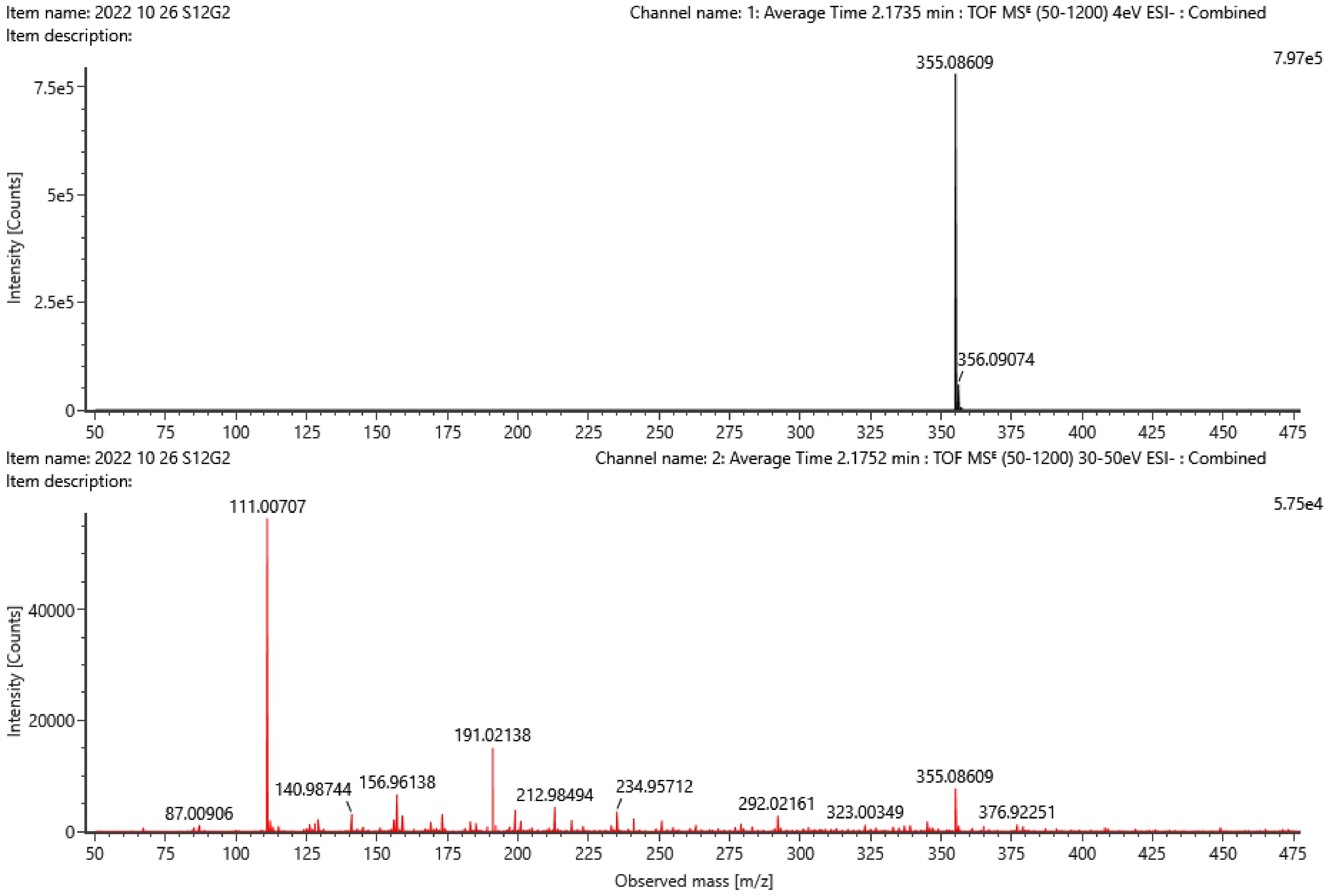

Figure S4: MS and MS/MS spectra of mannitol citrate, ESI$^-$

Table S2: Mass spectral information of mannitol stearate

**mannitol stearate**	
**molecular formula:** $C_{24}H_{48}O_7$	**estimated structure:** 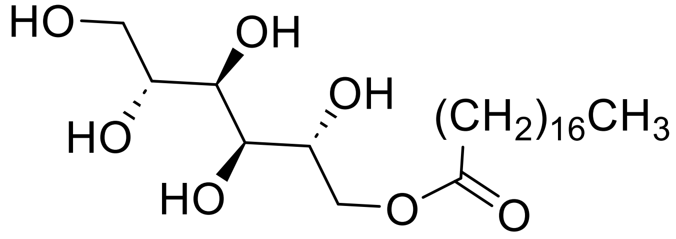
**retention time (min): 7.08**	

**negative ion mode**		**positive ion mode**	
**parent mass in MS spectra:**	**mass error (Δppm):**	**parent mass in MS spectra:**	**mass error (Δppm):**
**[M-H]$^-$** 447.3322<br>**[M+HCOO]$^-$** 493.3377	-1.10<br>-0.73	n.d.	-
**fragment ions in MS/MS spectra:**	**neutral loss:**	**fragment ions in MS/MS spectra:**	**neutral loss:**
283.265 (stearate)	$C_6H_{12}O_5$ (mannitol)	-	-

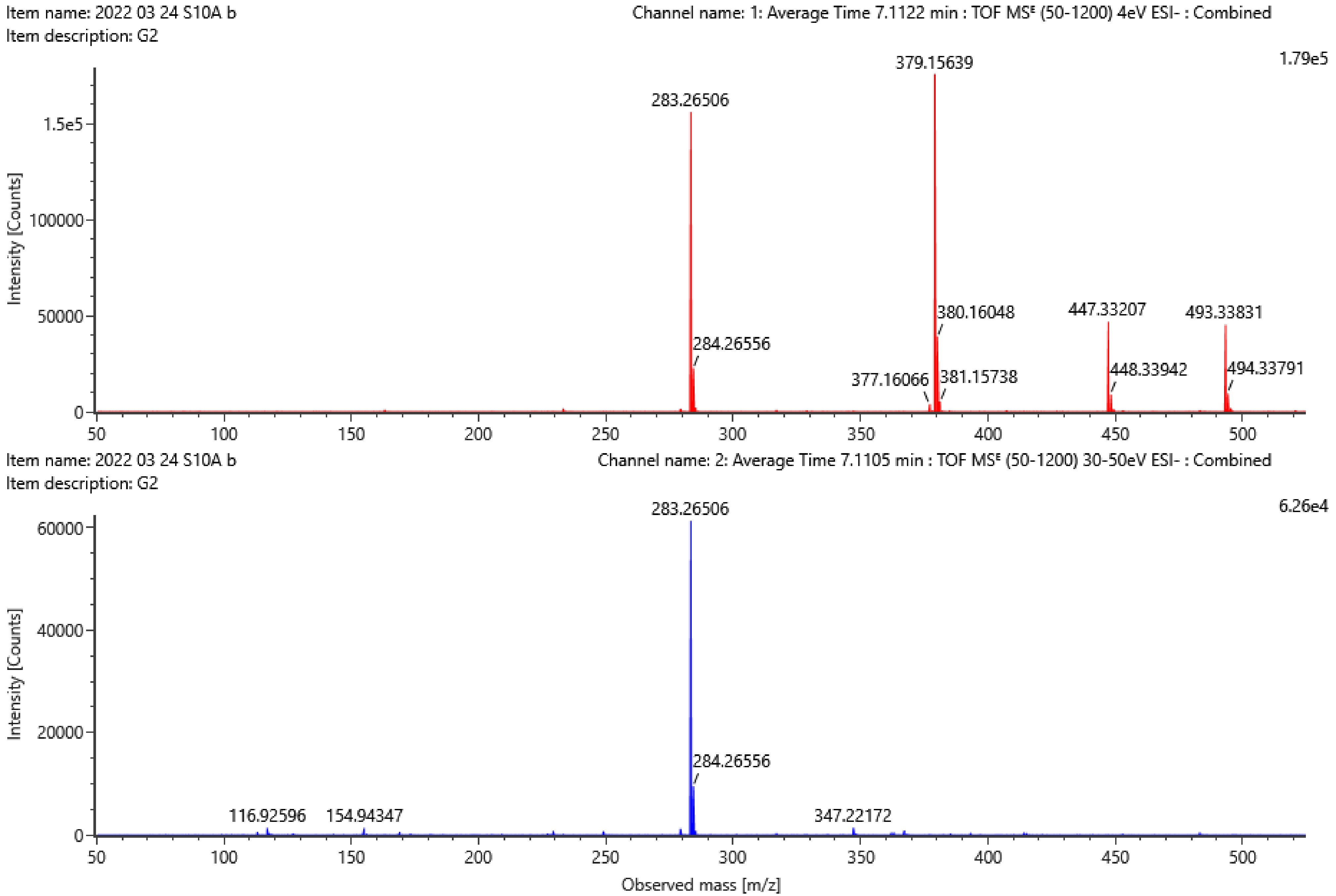

Figure S5: MS and MS/MS spectra of mannitol stearate, ESI$^-$

Table S3: Mass spectral information to mannitol palmitate

**mannitol palmitate**			
**molecular formula:** $C_{22}H_{44}O_7$	**estimated structure:** 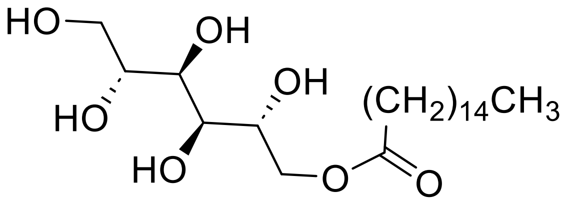		
**retention time (min): 6.57**			
**negative ion mode**		**positive ion mode**	
**parent mass in MS spectra:**	**mass error (Δppm):**	**parent mass in MS spectra:**	**mass error (Δppm):**
**[M-H]$^-$** 419.3009<br>**[M+HCOO]$^-$** 465.3065	-0.86<br>-0.95	n.d.	-
**fragment ions in MS/MS spectra:**	**neutral loss:**	**fragment ions in MS/MS spectra:**	**neutral loss:**
255.234 (palmitate)	$C_6H_{12}O_5$ (mannitol)	-	-

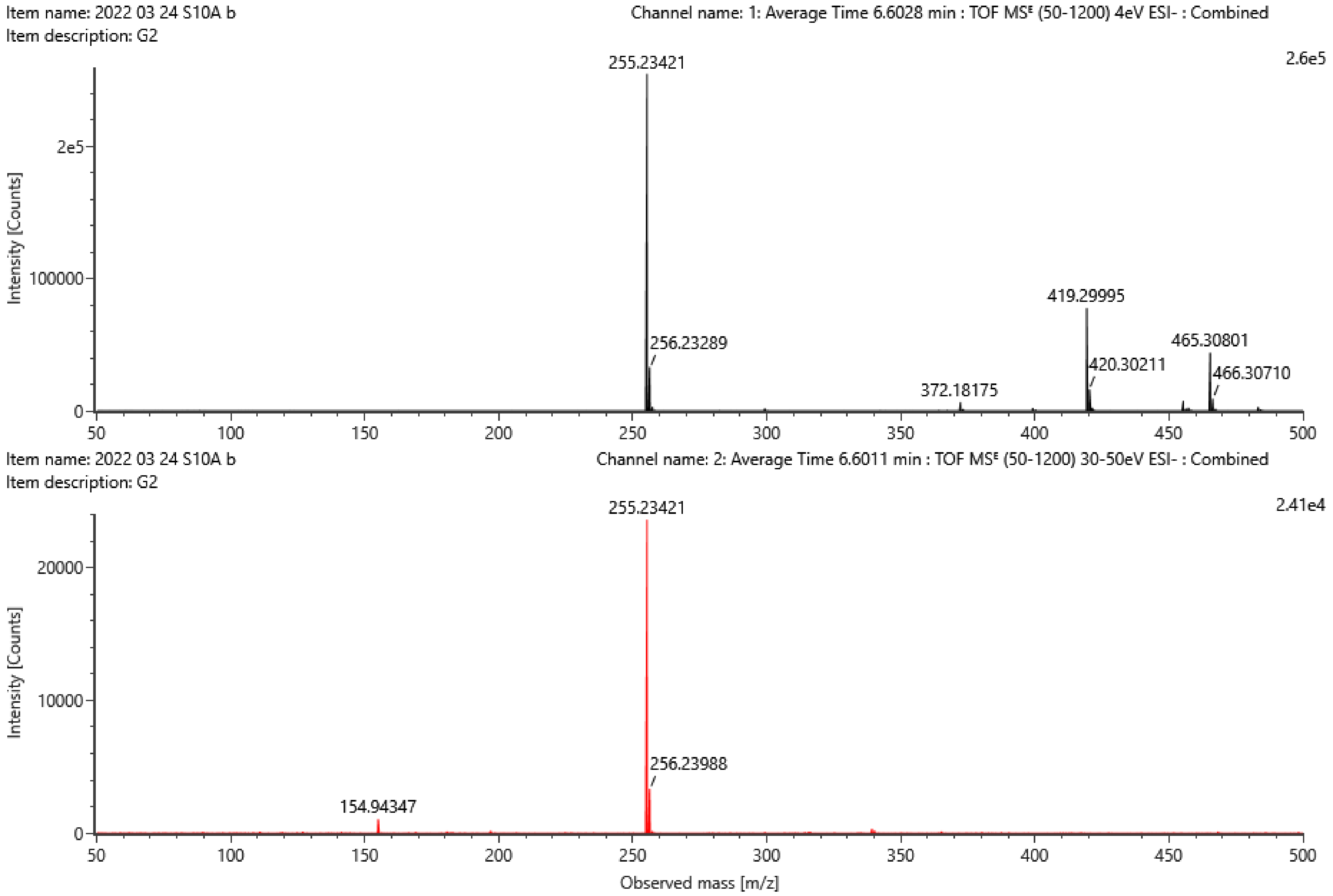

Figure S6: MS and MS/MS spectra of mannitol palmitate, ESI$^-$

Part B: phospholipids:

- **Table S4, Figure S7, S8: palmitoyl-lysophosphatidylcholine, LPC (16:0)**
- **Table S5, Figure S9, S10: linoleoyl-lysophosphatidylcholine, LPC (18:2)**
- **Table S6, Figure S11, S12: oleoyl-lysophosphatidylcholine, LPC (18:1)**
- **Table S7, Figure S13: palmitoyl-lysophosphatidylethanolamine, LPE (16:0)**
- **Table S8, Figure S14: linoleoyl-lysophosphatidylethanolamine, LPE (18:2)**
- **Table S9, Figure S15: oleoyl-lysophosphatidylethanolamine, LPE (18:1)**

Table S4: Mass spectral information for detected lysophosphatidylcholine C16:0

**LPC(16:0) palmitoyl-lysophosphatidylcholine**			
**molecular formula:** $C_{24}H_{50}NO_7P$<br>**retention time (min): 5.95 and 6.08**	**estimated structure:** 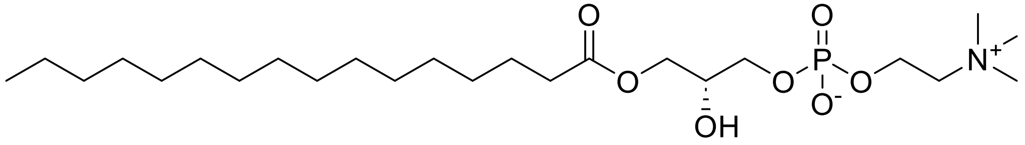		
**negative ion mode**		**positive ion mode**	
**parent mass in MS spectra:**	**mass error (Δppm):**	**parent mass in MS spectra:**	**mass error (Δppm):**
**[M+HCOO]$^-$** 540.3304<br>**[M-CH_3]$^-$** 480.3090	- 0.54<br>- 1.23	**[M+H]$^+$** 496.3405<br>[M+Na]$^+$ 518.3221	1.43<br>0.59
**fragment ions in MS/MS spectra:**	**neutral loss:**	**fragment ions in MS/MS spectra:**	**neutral loss:**
409.2363 ($C_{19}H_{38}O_7P$)<br>391.2249 ($C_{19}H_{36}O_6P$)<br>**255.2330 ($C_{16}H_{31}O_2$, palmitate)***<br>242.0795 ($C_7H_{17}NO_6P$)<br>224.0690 ($C_7H_{15}NO_5P$)<br>168.0424 ($C_4H_{11}NO_4P$)<br>152.9952 ($C_3H_6O_5P$)<br>78.9579 (PO_3)	$C_5H_{12}N$ (choline)<br>$C_5H_{14}NO$ (choline, H_2O)<br>**$C_8H_{19}NO_5P$ (glycerophosphocholine)**<br>$C_{16}H_{31}O$ (palmitate) + CH_3<br>$C_{16}H_{32}O_2$ (palmitate) + CH_3 + H_2O<br>$C_{20}H_{39}O_3$ (palmitate + CH_3 + glycerol)<br>$C_{21}H_{44}NO_2$ (palmitate + choline)<br>$C_{24}H_{50}NO_4$ (palmitate + glycerol + choline)	478.3295 ($C_{24}H_{49}NO_6P$)<br>313.2743 ($C_{19}H_{37}O_3$)<br>**184.0740 ($C_5H_{15}NO_4P$, phosphocholine head)**<br>124.9997 ($C_2H_6O_4P$)<br>104.1071 ($C_5H_{14}NO$)<br>86.0964 ($C_5H_{12}N$)	H_2O<br>$C_5H_{14}NO_4P$<br>**$C_{19}H_{36}O_3$ (palmitate + glycerol)**<br>$C_{22}H_{35}NO_3$<br>$C_{19}H_{35}NO_3P$<br>$C_{19}H_{37}NO_4P$

*bold = the most abundant fragment

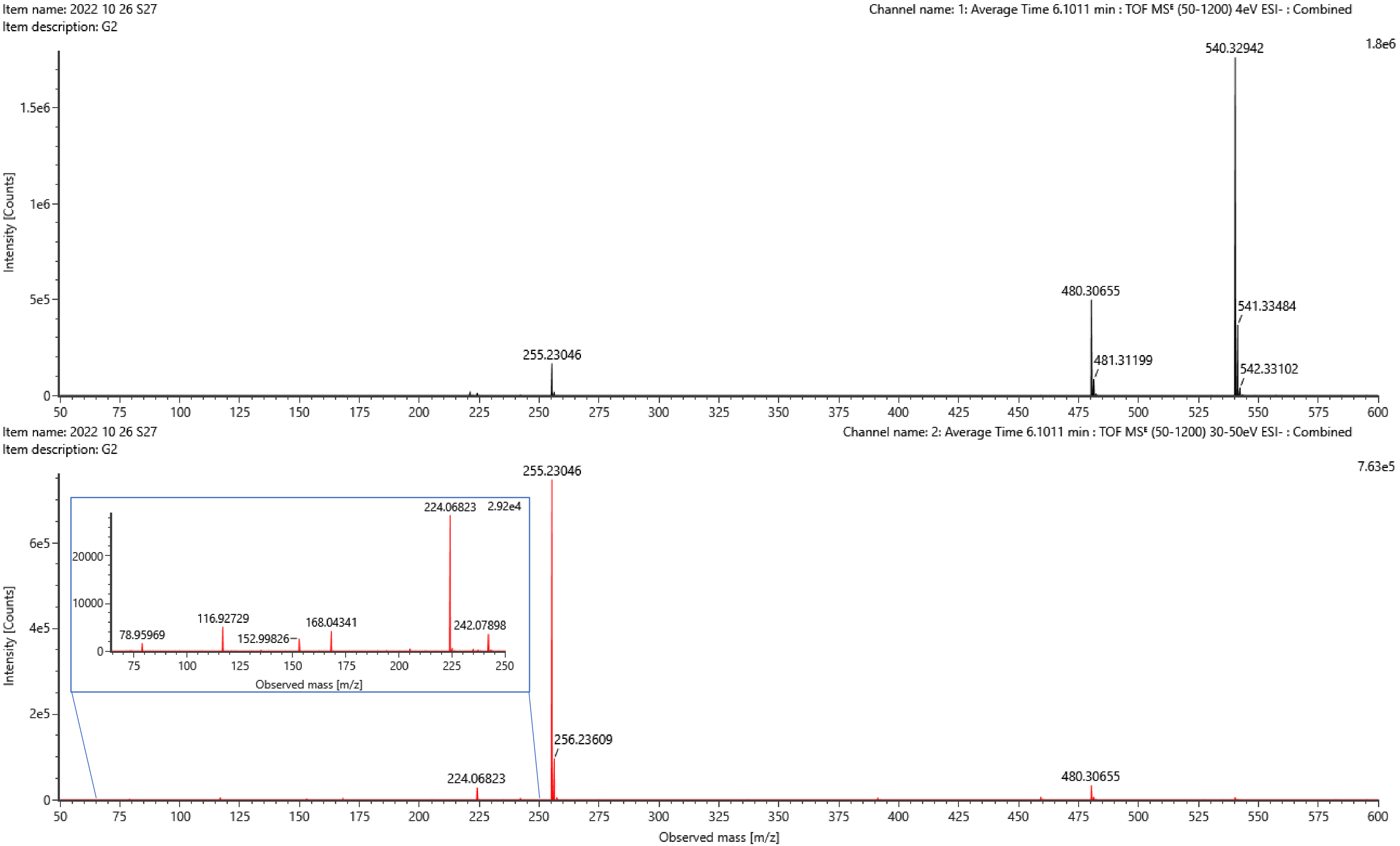

Figure S7: MS and MS/MS spectra of detected phospholipid LPC (16:0), ESI$^-$

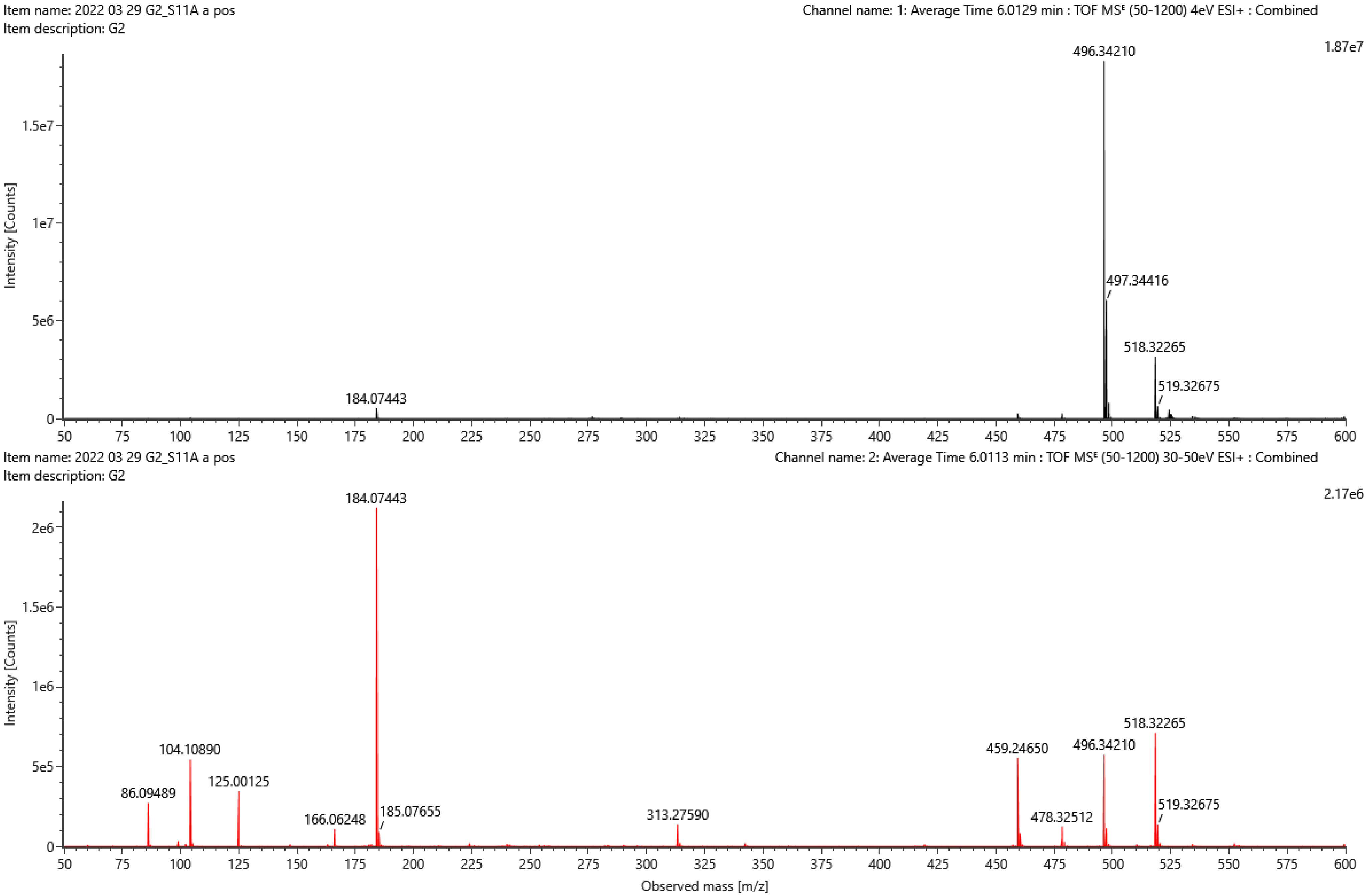

Figure S8: MS and MS/MS spectra of detected phospholipid LPC (16:0), ESI$^+$

Table S5: Mass spectral information to detected lysophosphatidylcholine C18:2

**LPC(18:2) linoleoyl-lysophosphatidylcholine**			
**Molecular formula:** $C_{26}H_{50}NO_7P$<br>**Retention time (min): 5.74; 5.84**	**estimated structure:** 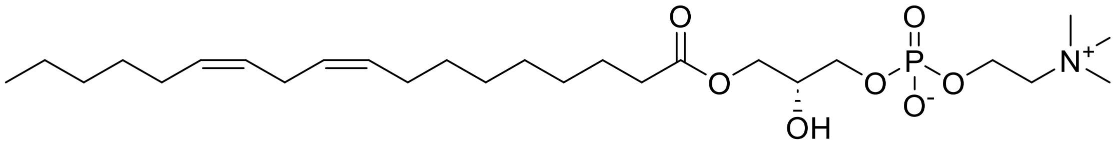		
**negative ion mode**		**positive ion mode**	
**parent mass in MS spectra:**	**mass error (Δppm):**	**parent mass in MS spectra:**	**mass error (Δppm):**
**[M+HCOO]$^-$** 564.3303<br>**[M-CH$_3$]$^-$** 504.3090	- 0.69<br>- 1.17	**[M+H]$^+$** 520.3406<br>[M+Na]$^+$ 542.3222	1.55<br>0.75
**fragment ions in MS/MS spectra:**	**neutral loss:**	**fragment ions in MS/MS spectra:**	**neutral loss:**
**279.2322 ($C_{18}H_{31}O_2$, linoleate)***<br>242.0795 ($C_7H_{17}NO_6P$)<br>224.0690 ($C_7H_{15}NO_5P$)<br>168.0424 ($C_4H_{11}NO_4P$)<br>152.9952 ($C_3H_6O_5P$)<br>78.9579 (PO_3)	**$C_8H_{19}NO_5P$ (glycerophosphocholine)**<br>$C_{18}H_{31}O$ (linoleate), CH_3<br>$C_{18}H_{31}O$ (linoleate), CH_3 + H_2O<br>$C_{22}H_{39}O_3$ (linoleate + CH_3 + glycerol)<br>$C_{23}H_{44}NO_2$ (linoleate + choline)<br>$C_{26}H_{50}NO_4$ (linoleate + glycerol + choline)	502.3302 ($C_{26}H_{49}NO_6P$)<br>337.2741 ($C_{21}H_{37}O_3$)<br>**184.0740** ($C_5H_{15}NO_4P$, phosphocholine head)<br>124.9997 ($C_2H_6O_4P$)<br>104.1075 ($C_5H_{14}NO$)<br>86.0964 ($C_5H_{12}N$)	H_2O<br>$C_5H_{14}NO_4P$ (phosphocholine)<br>**$C_{21}H_{36}O_3$ (linoleate, glycerol)**<br>$C_{24}H_{35}NO_3$<br>$C_{21}H_{35}NO_3P$<br>$C_{21}H_{37}NO_4P$

*bold = the most abundant fragment

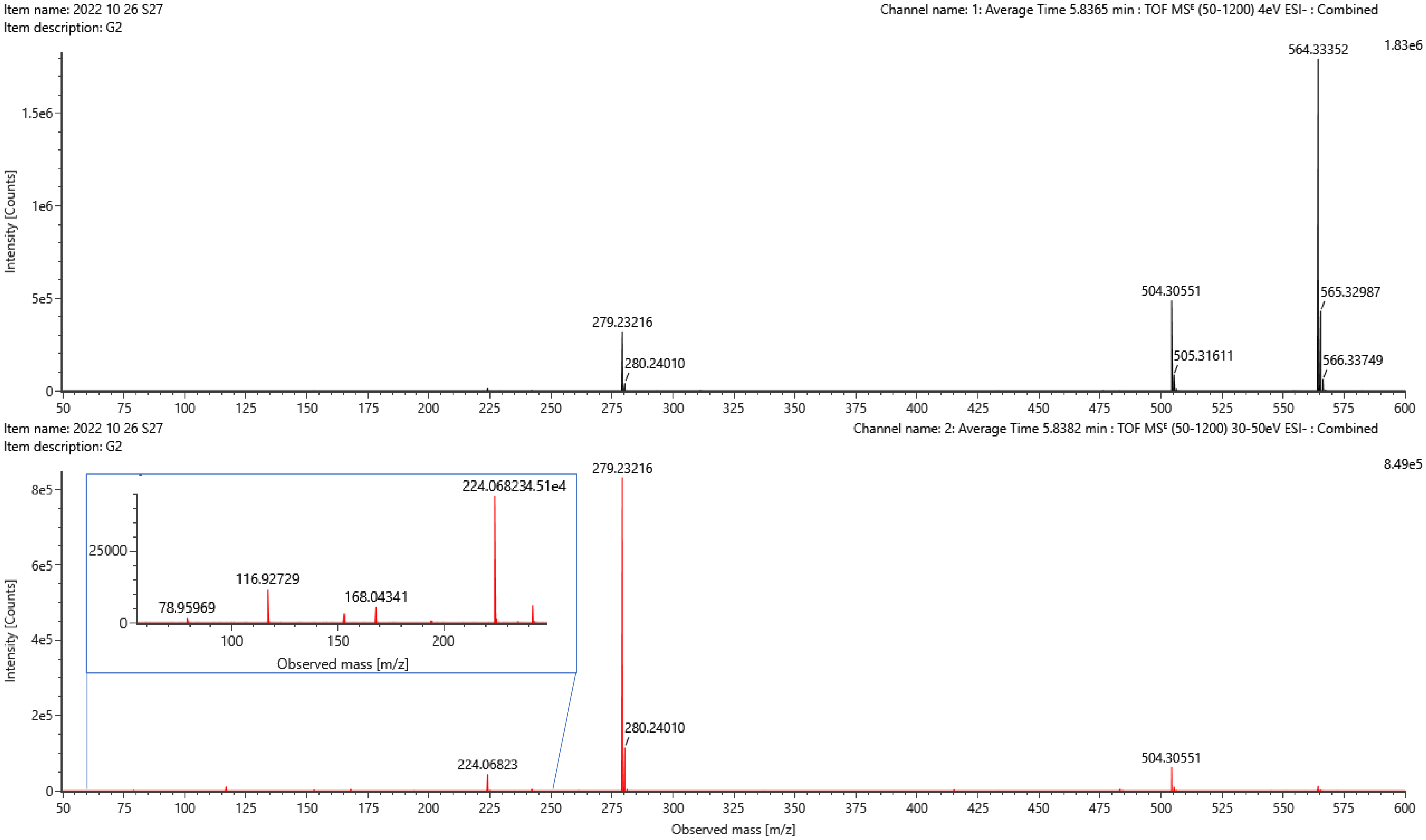

Figure S9: MS and MS/MS spectra of detected phospholipid LPC (18:2), ESI⁻

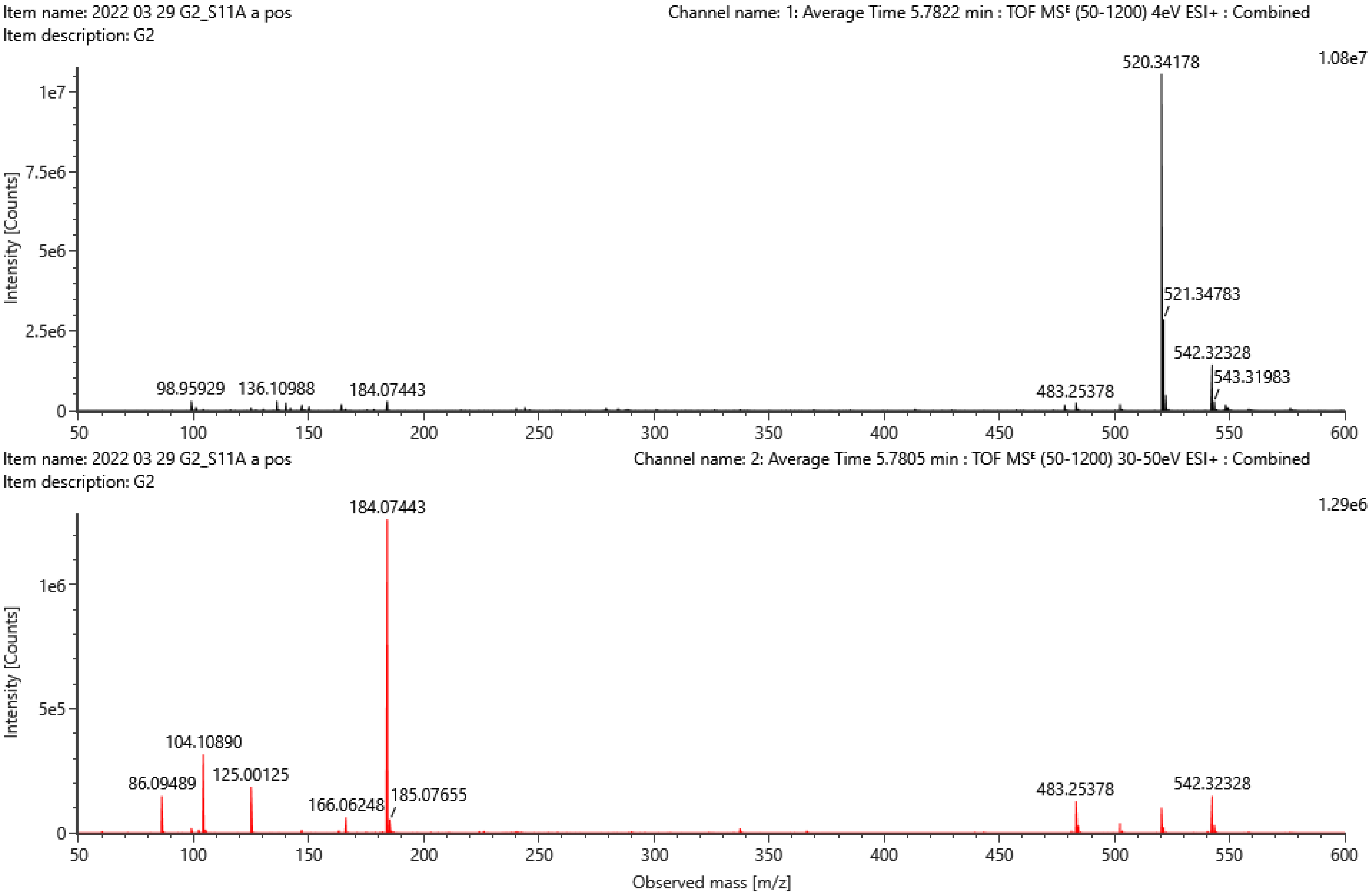

Figure S10: MS and MS/MS spectra of detected phospholipid LPC (18:2), ESI$^+$

Table S6: Mass spectral information to detected lysophosphatidylcholine C18:1

**LPC(18:2) oleoyl-lysophosphatidylcholine**			
**Molecular formula:** $C_{26}H_{52}NO_7P$	**estimated structure:** 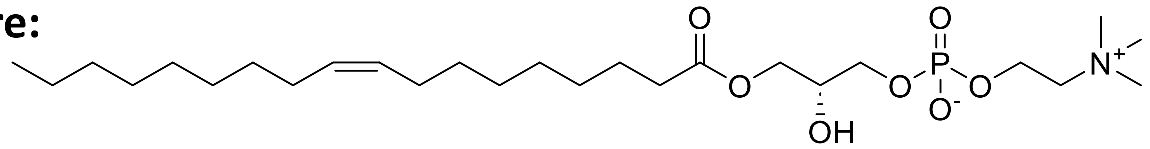		
**Retention time (min):** 5.74; **5.84**			
**negative ion mode**		**positive ion mode**	
**parent mass in MS spectra:**	**Mass error (Δppm)**	**parent mass in MS spectra:**	**mass error (Δppm)**
**$[M+HCOO]^-$** 566.3463<br>**$[M-CH_3]^-$** 506.3247	- 0.78<br>0.00	**$[M+H]^+$** 522.3559<br>$[M+Na]^+$ 544.3377	0.88<br>0.47
**Fragment ions in MS/MS spectra:**	**Neutral loss**	**Fragment ions in MS/MS spectra:**	**Neutral loss**
**281.2479 (oleate)**<br><br>242.0795 ($C_7H_{17}NO_6P$)<br>224.0690 ($C_7H_{15}NO_5P$)<br>168.0424 ($C_4H_{11}NO_4P$)<br><br>152.9952 ($C_3H_6O_5P$)<br>78.9579 (PO_3)	**$C_8H_{19}NO_5P$ (glycerophosphocholine)**<br>$C_{18}H_{33}O$ (oleate), CH_3<br>$C_{18}H_{33}O$ (oleate), CH_3, H_2O<br>$C_{22}H_{41}O_3$ (oleate, CH_3, glycerol)	504.3476 ($C_{26}H_{51}NO_6P$)<br>339.2892 ($C_{21}H_{39}O_3$)<br>**184.0740 ($C_5H_{15}NO_4P$, phosphocholine head)**<br>124.9997 ($C_2H_6O_4P$)<br>104.1071 ($C_5H_{14}NO$)<br>86.0949 ($C_5H_{12}N$)	H_2O<br>$C_5H_{14}NO_4P$ (phosphocholine)<br>$C_{21}H_{38}O_3$ (oleate + glycerol)<br><br>$C_{24}H_{47}NO_3$<br>$C_{24}H_{37}NO_3P$<br>$C_{24}H_{39}NO_4P$

*bold = the most abundant fragment

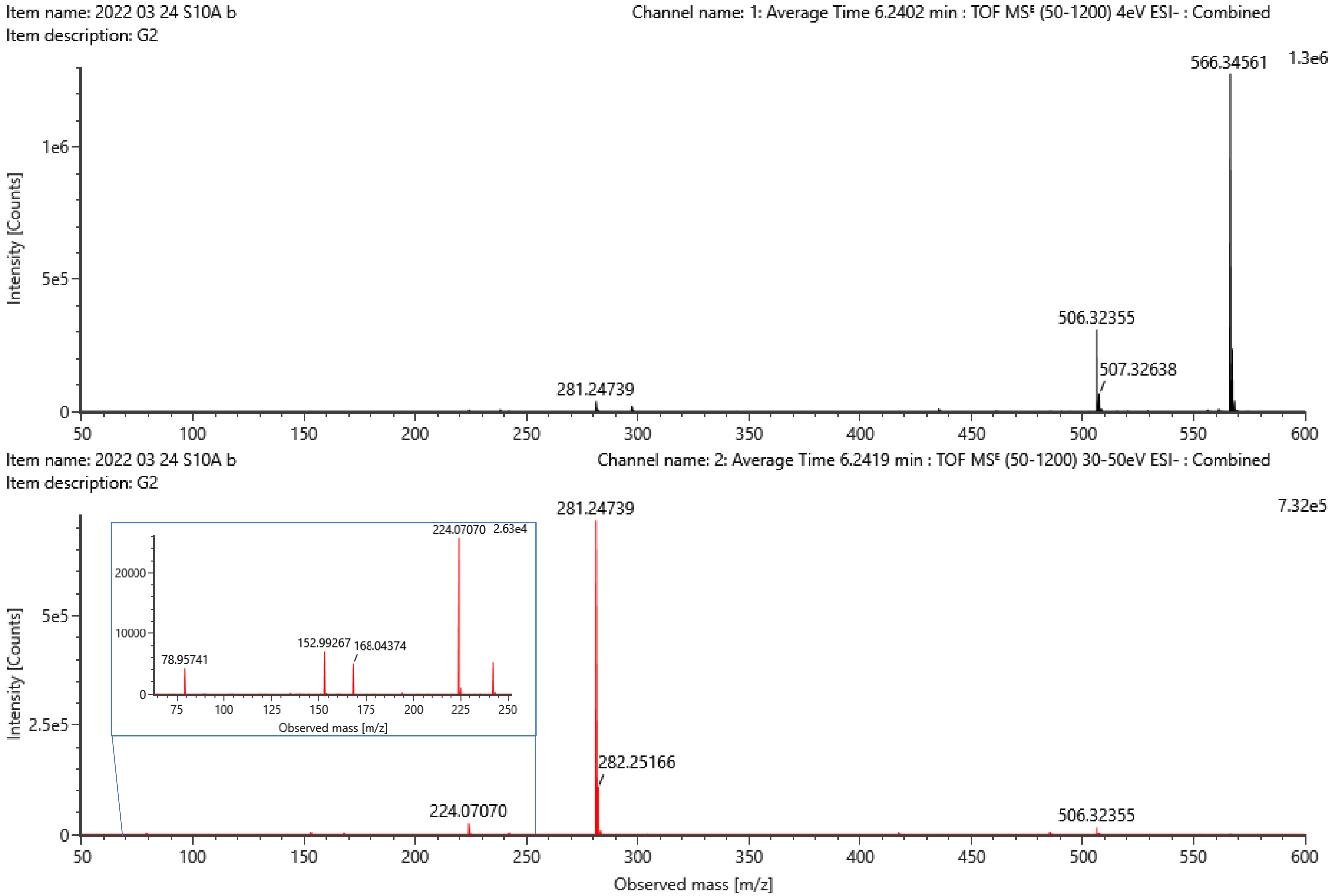

Figure S11: MS and MS/MS spectra of detected phospholipid LPC (18:1), ESI$^-$

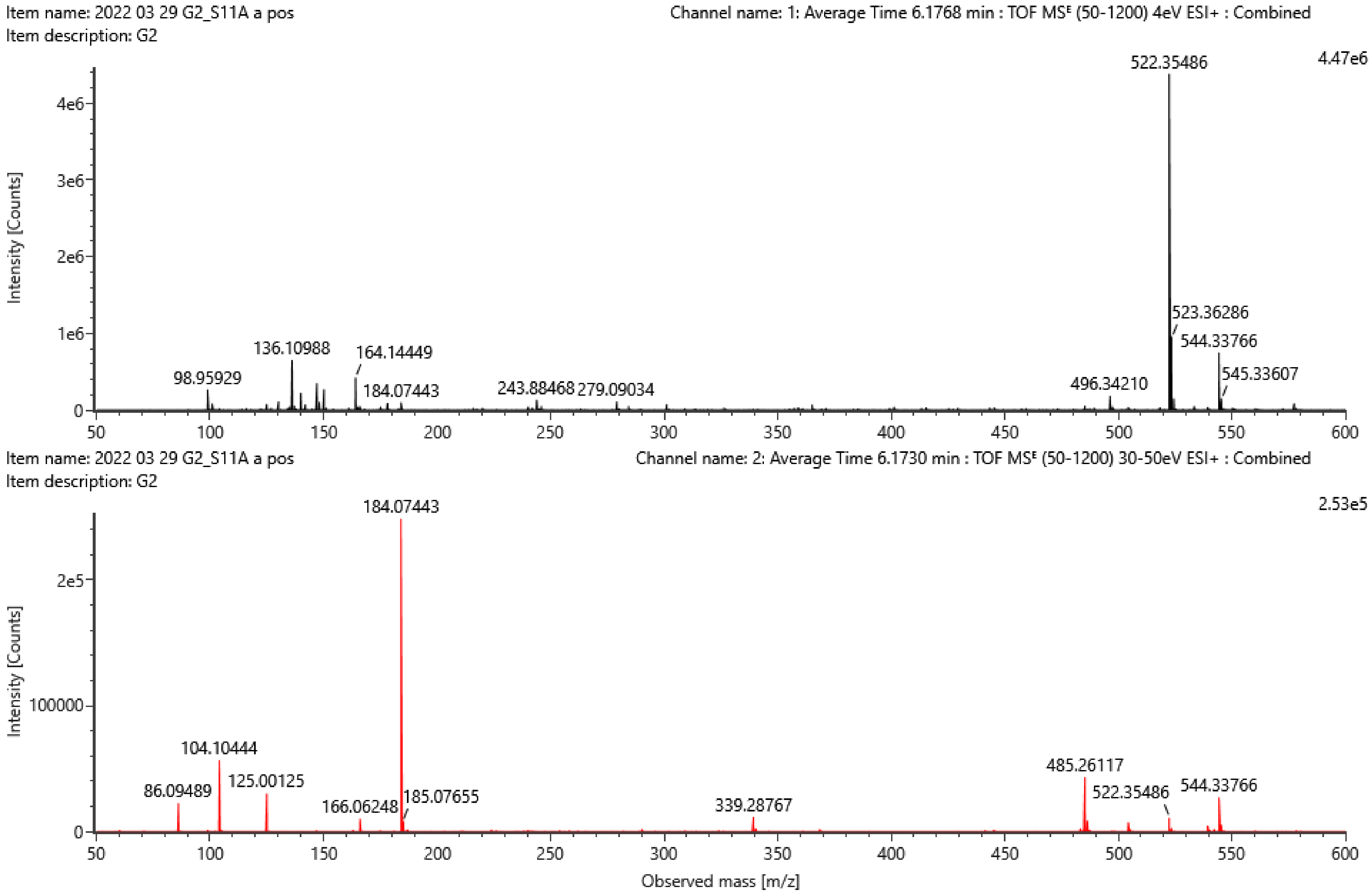

Figure S12: MS and MS/MS spectra of detected phospholipid LPC (18:1), ESI$^+$

Table S7: Mass spectral information to detected lysophosphatidylethanolamine C16:0

**LPE(16:0) palmitoyl-lysophosphatidylethanolamine**			
**molecular formula:** $C_{21}H_{44}NO_7P$	**estimated structure:** 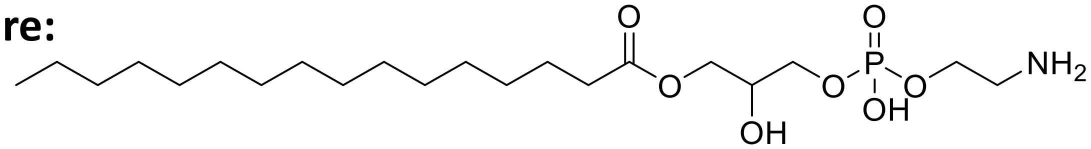		
**retention time (min):** 5.88; **6.00**			
**negative ion mode**		**positive ion mode**	
**parent mass in MS spectra:**	**mass error (Δppm):**	**parent mass in MS spectra:**	**mass error (Δppm):**
**$[M\text{-}H]^-$** 452.2780	- 0.62	**$[M+H]^+$** 454.2933<br>$[M+Na]^+$ 476.2751	1.03<br>0.63
**fragment ions in MS/MS spectra:**	**neutral loss:**	**fragment ions in MS/MS spectra:**	**neutral loss:**
**255.2342 ($C_{16}H_{31}O_2$, palmitate)***<br>196.0407 ($C_5H_{11}NO_5P$)	**$C_5H_{12}NO_5P$ (glycerophosphocholine)**<br>$C_5H_{14}NO$ (palmitate)	n.a.	-

*bold = the most abundant fragment

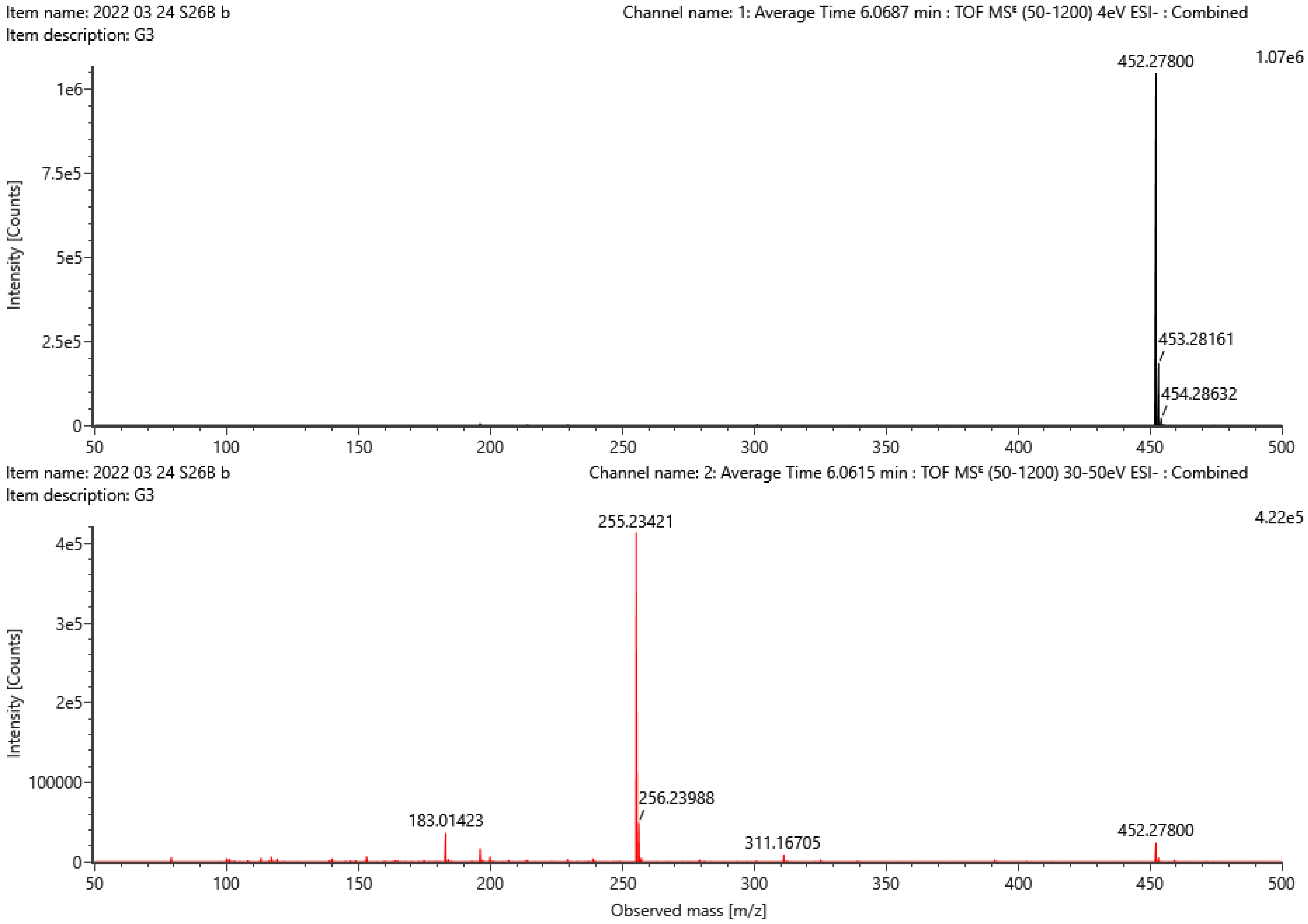

Figure S13: MS and MS/MS spectra of detected phospholipid LPE (16:0), ESI^-

Table S8: Mass spectral information to detected lysophosphatidylethanolamine C18:2

**LPE(18:2) palmitoyl-lysophosphatidylethanolamine**			
**molecular formula:** $C_{23}H_{44}NO_7P$	**estimated structure:** 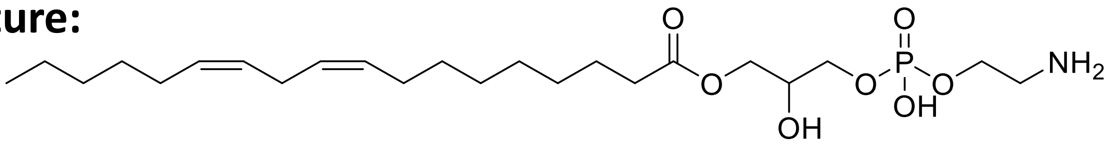		
**retention time (min): 5.78**			
**negative ion mode**		**positive ion mode**	
**parent mass in MS spectra:**	**mass error (Δppm):**	**parent mass in MS spectra:**	**mass error (Δppm):**
**$[M\text{-}H]^-$** 476.2779	- 0.63	**$[M+H]^+$** 478.2934<br>$[M+Na]^+$ 500.2749	1.21<br>0.08
**fragment ions in MS/MS spectra:**	**neutral loss:**	**fragment ions in MS/MS spectra:**	**neutral loss:**
**279.2369 ($C_{18}H_{31}O_2$, linoleate)***<br>196.0407 ($C_5H_{11}NO_5P$)	**$C_5H_{12}NO_5P$ (glycerophosphocholine)**<br>$C_{18}H_{31}O_2$ (linoleate)	n.a.	-

*bold = the most abundant fragment

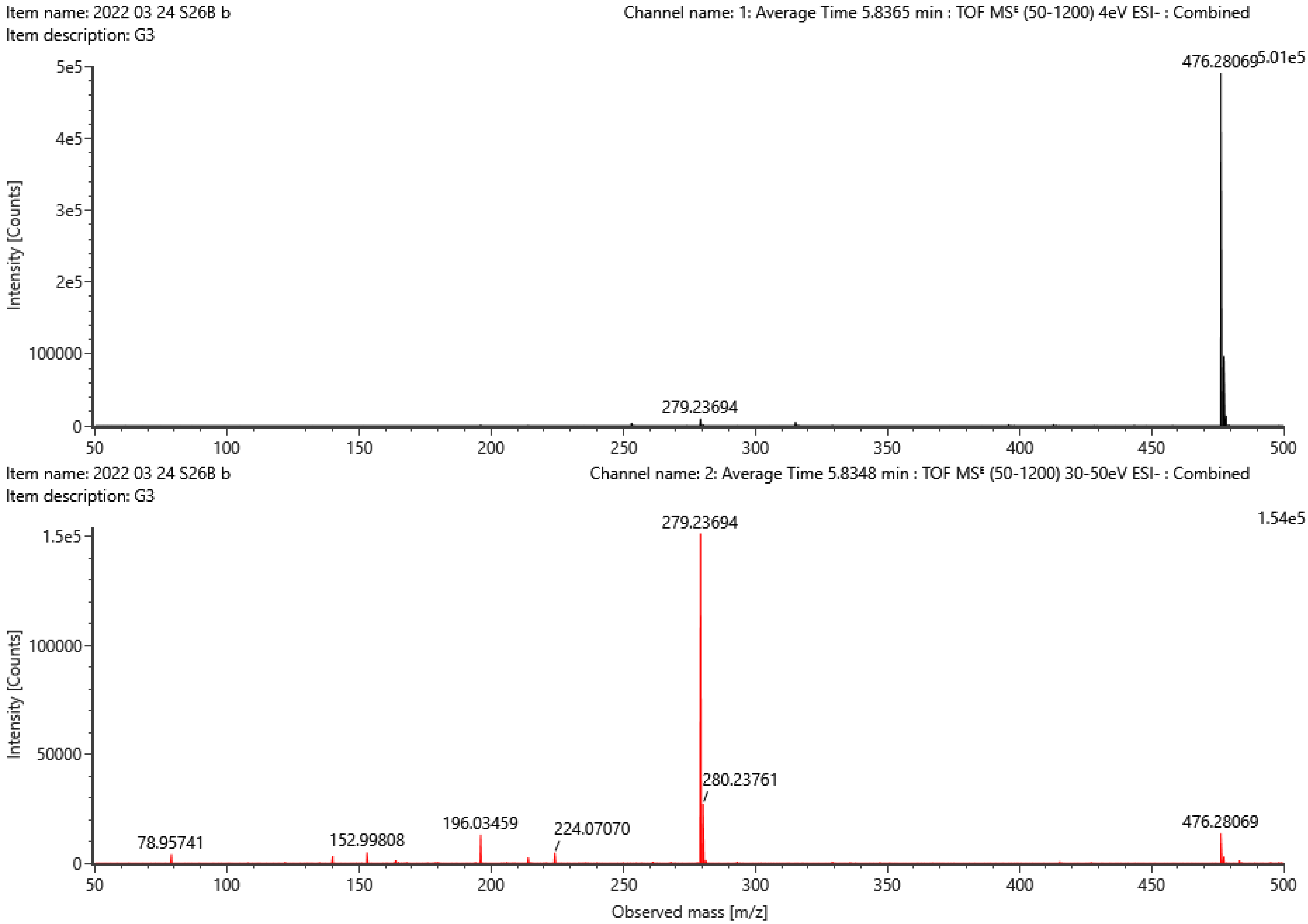

Figure S14: MS and MS/MS spectra of detected phospholipid LPE (18:2), ESI^-

Table S9: Mass spectral information to detected lysophosphatidylethanolamine C18:1

**LPE(18:1) oleoyl-lysophosphatidylethanolamine**			
**molecular formula:** $C_{23}H_{46}NO_7P$	**estimated structure:** 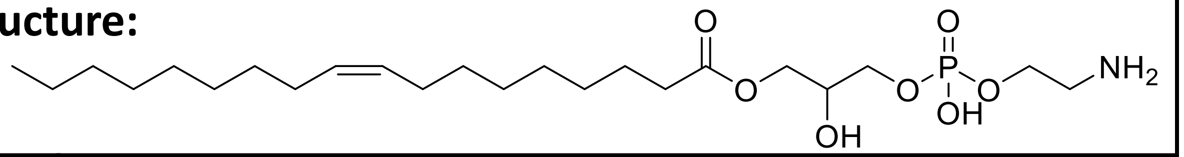		
**retention time (min): 6.16**			
**negative ion mode**		**positive ion mode**	
**parent mass in MS spectra:**	**mass error (Δppm):**	**parent mass in MS spectra:**	**mass error (Δppm):**
**$[M\text{-}H]^-$ 478.2933**	- 1.18	**$[M+H]^+$** 480.3086<br>$[M+Na]^+$ 502.2904	0.29<br>-0.28
**fragment ions in MS/MS spectra:**	**neutral loss:**	**fragment ions in MS/MS spectra:**	**neutral loss:**
**281.2474 (oleate)**	**$C_5H_{12}NO_5P$ (glycerophosphocholine)**		

*bold = the most abundant fragment

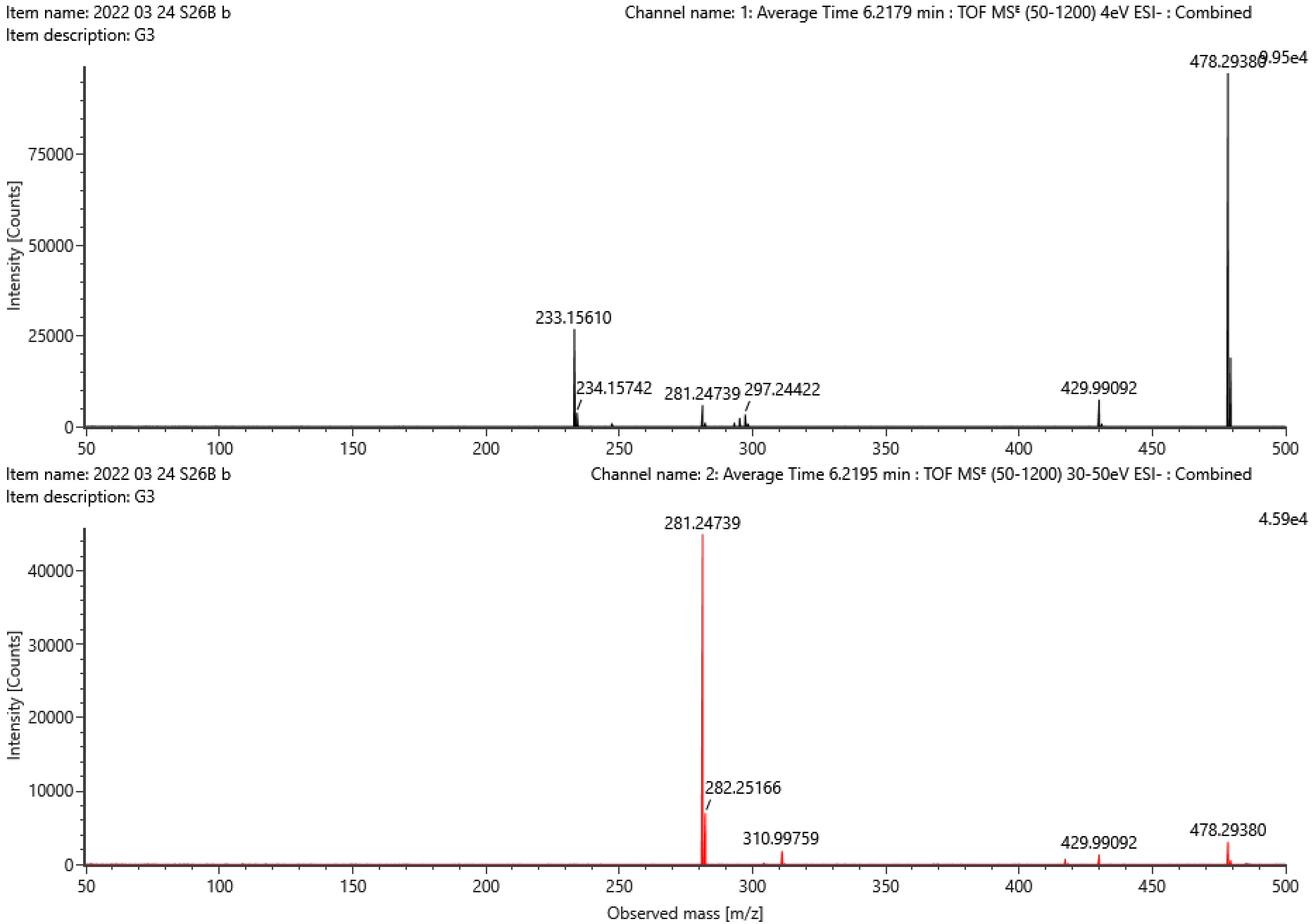

Figure S15: MS and MS/MS spectra of detected phospholipid LPE (18:1), ESI^-

Part C: Levothyroxine-derived impurities:

- **Figure S16:** Examples of chromatograms comparing DMSO and 50% MeOH extracts
- **Table S10, Figure S17, S18:** impurity no. 1: levothyroxine lactose adduct
- **Table S11, Figure S19, S20:** impurity no. 2: Liothyronine
- **Table S12, Figure S21:** impurity no. 3: 2-Hydroxy-2-(4-(4-hydroxy-3,5-diiodophenoxy)-3,5-diiodophenyl) acetic acid
- **Table S13, Figure S22, S23: impurity no. 4:** 2-(4-(4-hydroxy-3,5-diiodophenoxy)-3,5-diiodophenyl) acetamide
- **Table S14, Figure S24: impurity no. 5:** 2-(4-(4-hydroxy-3,5-diiodophenoxy)-3,5-diiodophenyl)-2-oxoacetamide
- **Table S15, Figure S25: impurity no. 6:** 4-(4-hydroxy-3,5-diiodophenoxy)-3,5-diiodo benzoic acid
- **Table S16, Figure S26: impurity no. 7:** 4-(4-hydroxy-3,5-diiodophenoxy)-3,5-diiodo acetaldehyde
- **Table S17, Figure S27: impurity no. 8:** 4-(4-hydroxy-3,5-diiodophenoxy)-3,5-diiodo benzaldehyde
- **Table S18:** overview of relative intensities of levothyroxine-derived impurities

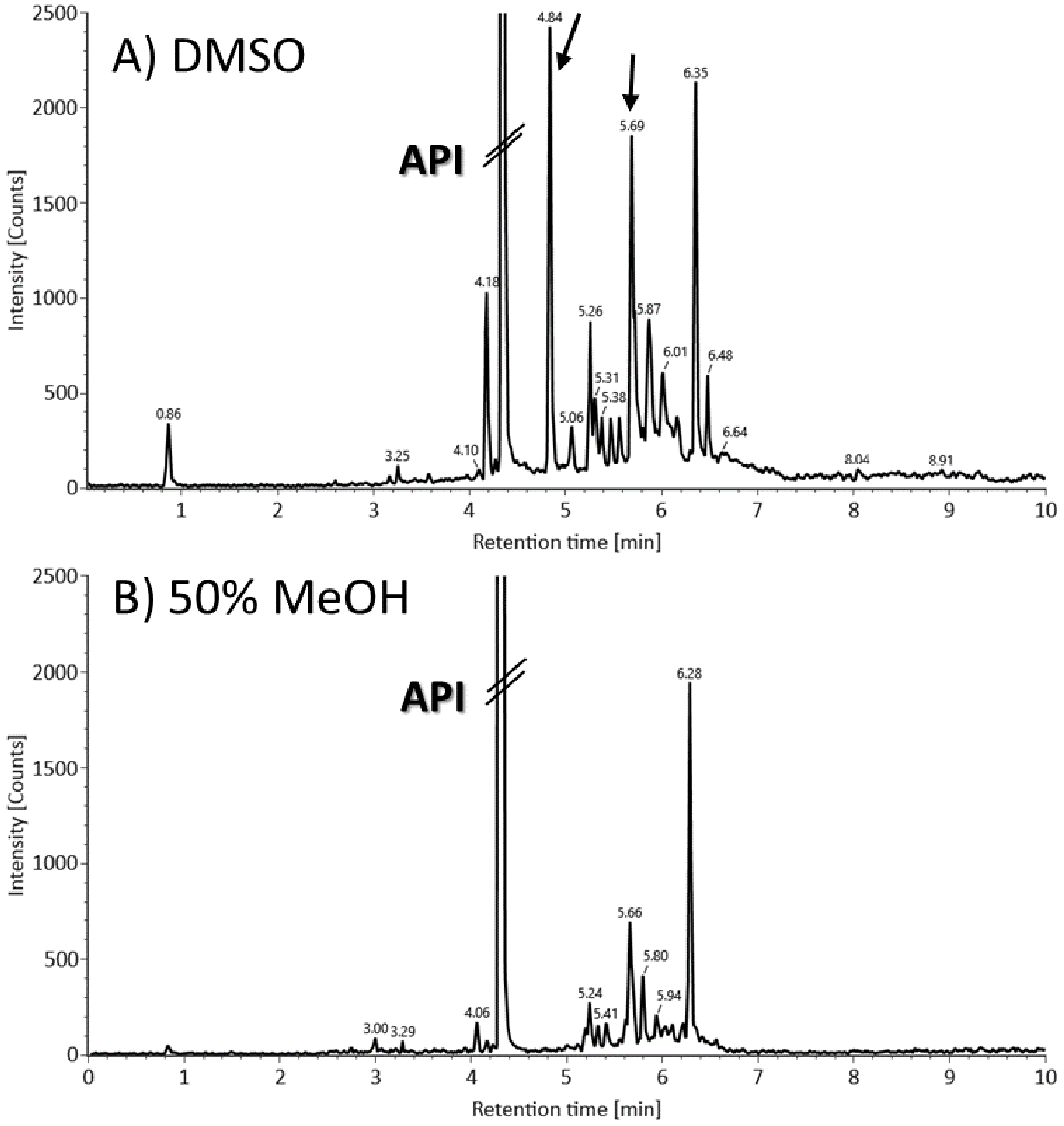

Figure 16: Examples of XIC chromatograms in ESI^- for fragment $[I]^-$ at *m/z* = 126.905: A) DMSO and B) 50% MeOH extracts of M4 tablets (arrows point to dominant peaks present only in the DMSO extract).

Table S10: Mass spectral information to levothyroxine impurity No. 1.

**No. 1: Levothyroxine lactose adduct (Maillard reaction product)**			
**Molecular formula: $C_{27}H_{31}I_4NO_{14}$**	**estimated structure:** 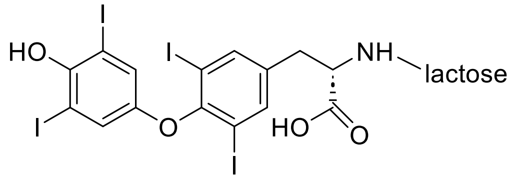		
**Retention time (min): 4.05**			
**Negative ion mode**		**Positive ion mode**	
**Measured mass [M-H]$^-$ (parent mass in MS spectra)**	**Mass error (Δppm)**	**Measured mass [M+H]$^+$ (parent mass in MS spectra):**	**Mass error (Δppm)**
1099.7849	-0.09	1101.8009	1.18
**fragment ions in MS/MS spectra:**	**neutral loss:**	**fragment ions in MS/MS spectra:**	**neutral loss:**
919.7132	$C_6H_{12}O_6$	**921.7370***	**$C_6H_{12}O_6$**
881.6767	$C_6H_{12}O_5 + 3H_2O$	903.7262	$C_6H_{12}O_6 + H_2O$
857.7182	$C_6H_{12}O_6 + CO_2 + H_2O$	875.7323	$C_6H_{12}O_6 + CO_2$
**815.7146***	**$CO_2 + C_8H_{16}O_8$**	789.6933	$C_{11}H_{20}O_{10}$
797.6953	$C_9H_{18}O_{11}$	777.6943	$C_{12}H_{22}O_{10}$
758.6522	$C_{12}H_{22}O_{10}$-NH-	743.6904	$C_{11}H_{22}O_{10} + CO_2$
741.6740	$C_{11}H_{22}O_{10} + CO_2$	731.6888	$C_{12}H_{22}O_{10} + CO_2$
574.7536	$C_{14}H_{24}NO_{12} + I$	702.6628	$C_{14}H_{24}NO_{12}$

*bold = the most abundant fragment

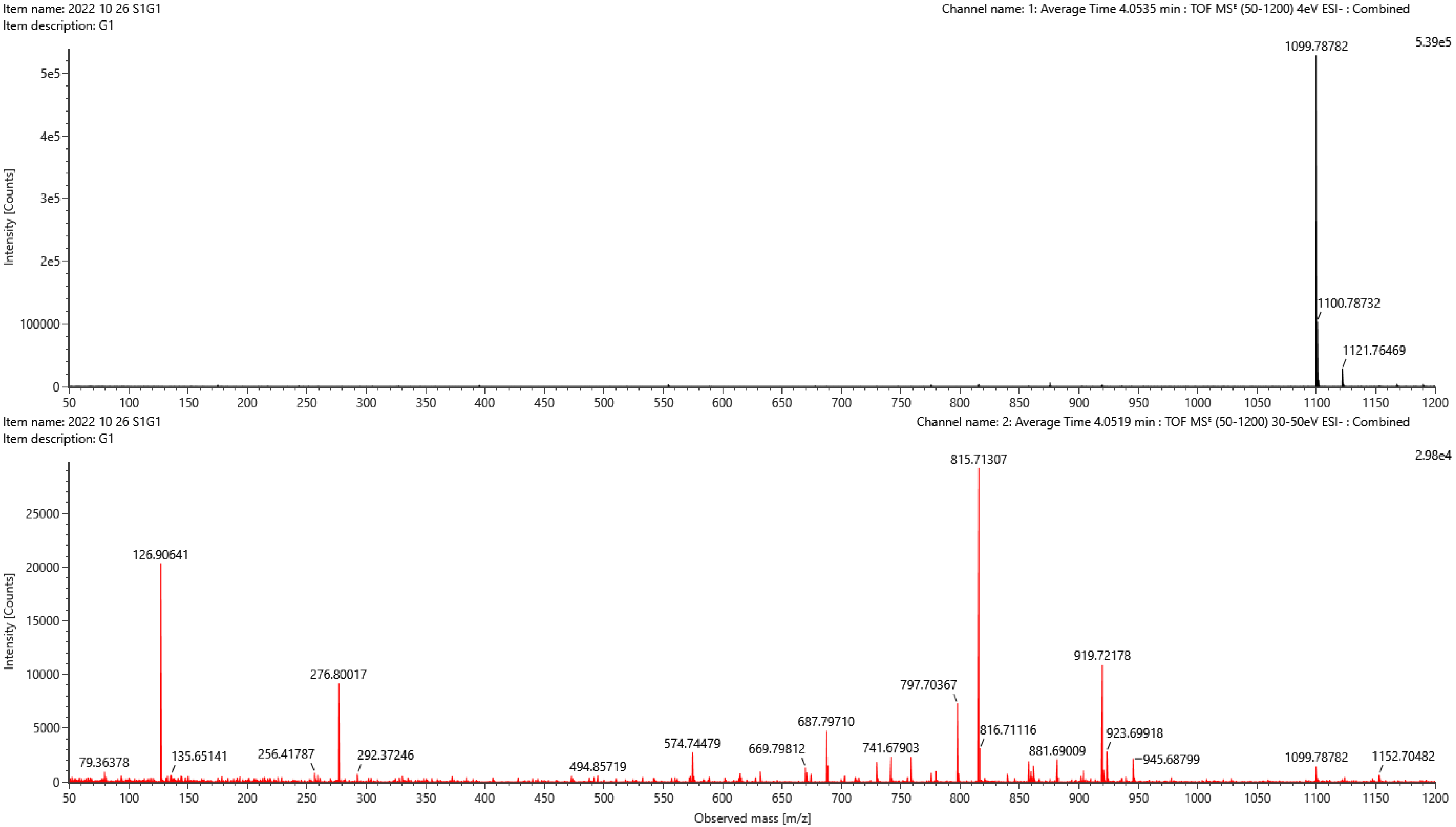

Figure S17: MS and MS/MS spectra of levothyroxine lactose adduct, ESI^-

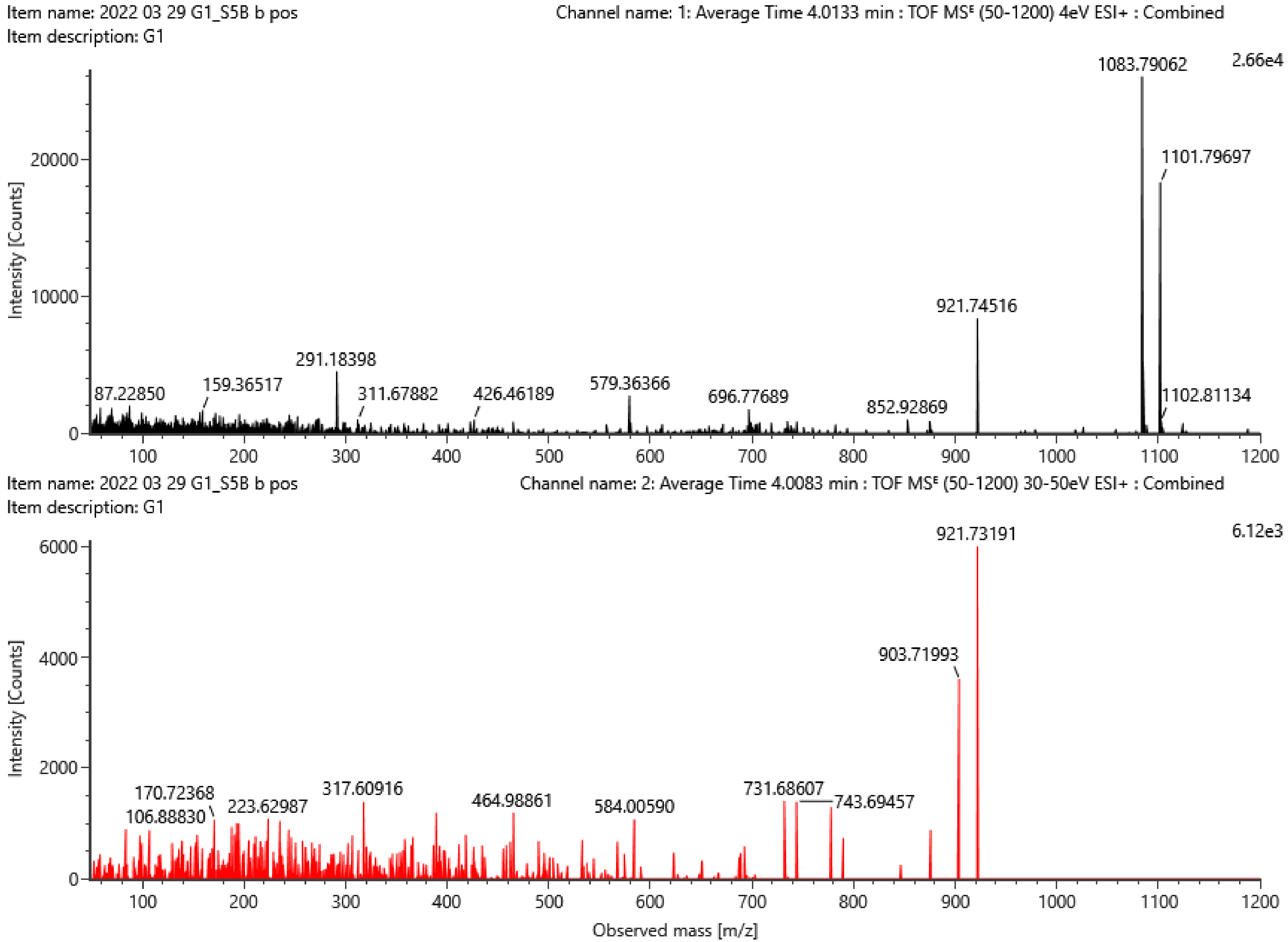

Figure S18: MS and MS/MS spectra of levothyroxine lactose adduct, ESI$^+$

Table S11: Mass spectral information to levothyroxine impurity No. 2.

**No. 2: Liothyronine (triiodothyronine)**			
**Molecular formula: $C_{15}H_{12}I_3NO_4$**	**estimated structure:** 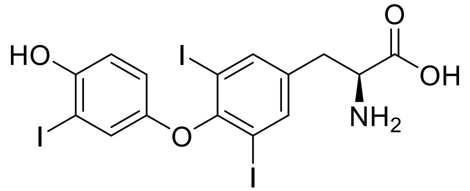		
**Retention time (min): 4.10**			
**Negative ion mode**		**Positive ion mode**	
**Measured mass $[M-H]^-$ (parent mass in MS spectra)**	**Mass error (Δppm)**	**Measured mass $[M+H]^+$ (parent mass in MS spectra):**	**Mass error (Δppm)**
649.7831	0.62	651.7982	2.30
**fragment ions in MS/MS spectra:**	**neutral loss:**	**fragment ions in MS/MS spectra:**	**neutral loss:**
**448.8576***	$I + C_2H_4NO_2$	605.7927<br>478.8866<br>450.8690	CO_2<br>$CO_2 + I$<br>$C_2H_4NO_2 + I$

*bold = the most abundant fragment

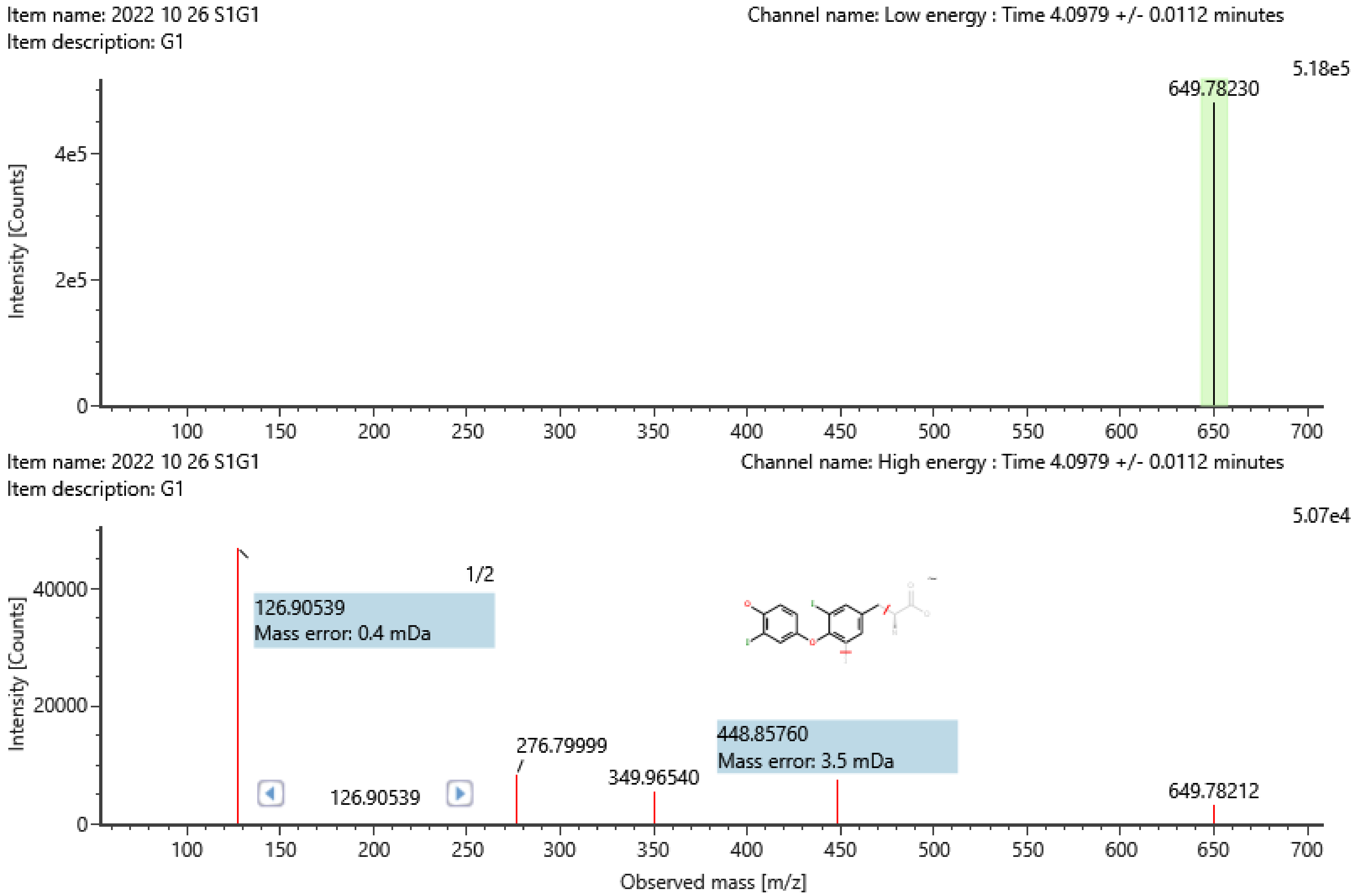

Figure S19: MS and MS/MS spectra of liothyronine, ESI$^-$

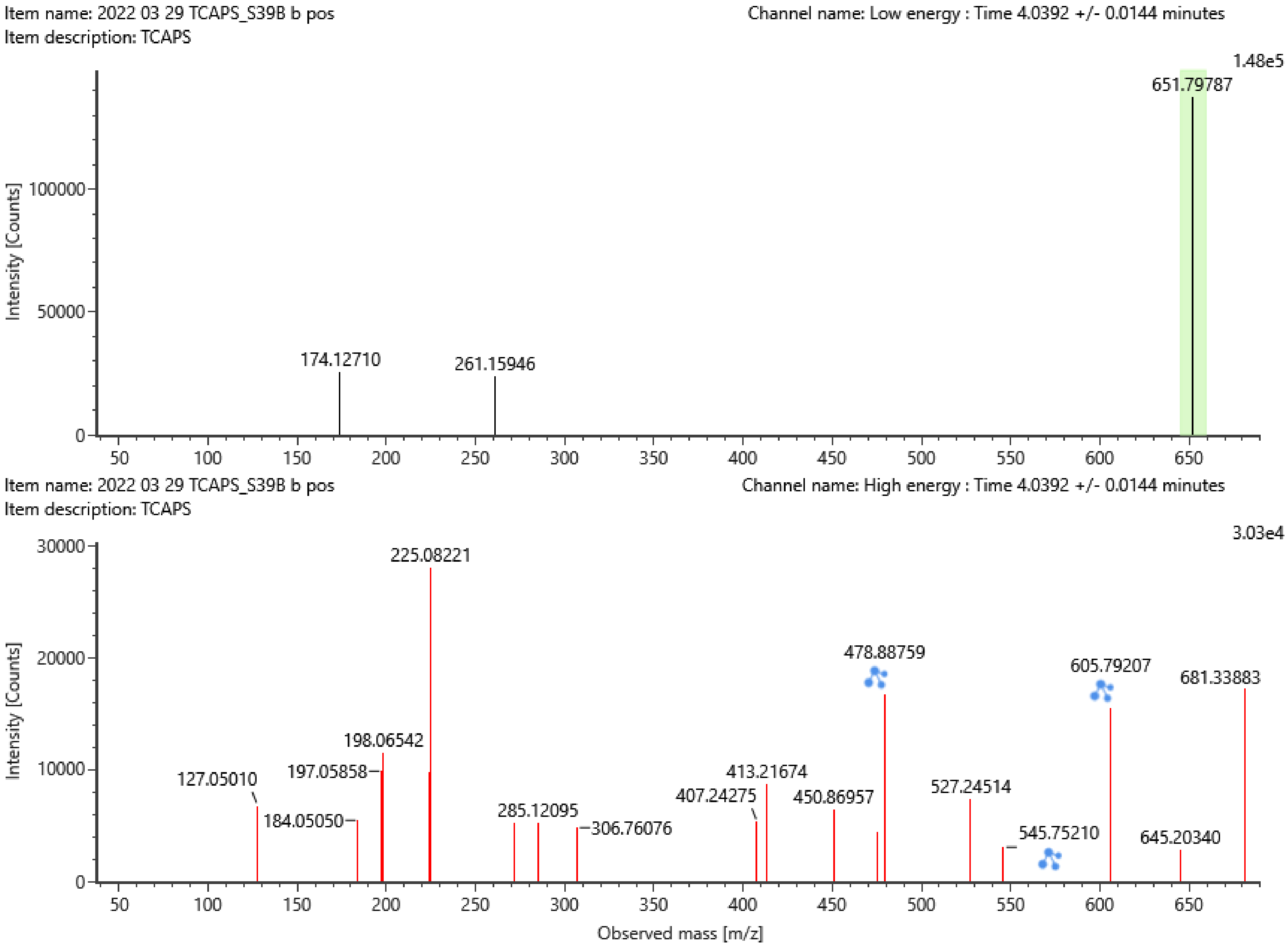

Figure S20: MS and MS/MS spectra of liothyronine, ESI⁺

Table S12: Mass spectral information to levothyroxine impurity No. 3.

**No. 3: 2-Hydroxy-2-(4-(4-hydroxy-3,5-diiodophenoxy)-3,5-diiodophenyl) acetic acid**			
**Molecular formula: $C_{14}H_8I_4O_5$**	**estimated structure:** 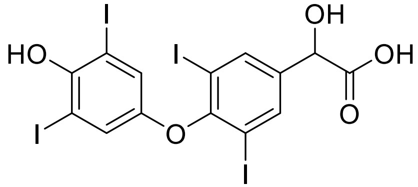		
**Retention time (min): 5.39**			
**Negative ion mode**		**Positive ion mode**	
**Measured mass [M-H]$^-$ (parent mass in MS spectra)**	**Mass error (Δppm)**	**Measured mass [M+H]$^+$ (parent mass in MS spectra):**	**Mass error (Δppm)**
762.6484	0.92	n.d.	-
**fragment ions in MS/MS spectra:**	**neutral loss:**	**fragment ions in MS/MS spectra:**	**neutral loss:**
744.6377<br>**617.7334***	H_2O<br>**H_2O + I**	-	-

*bold = the most abundant fragment

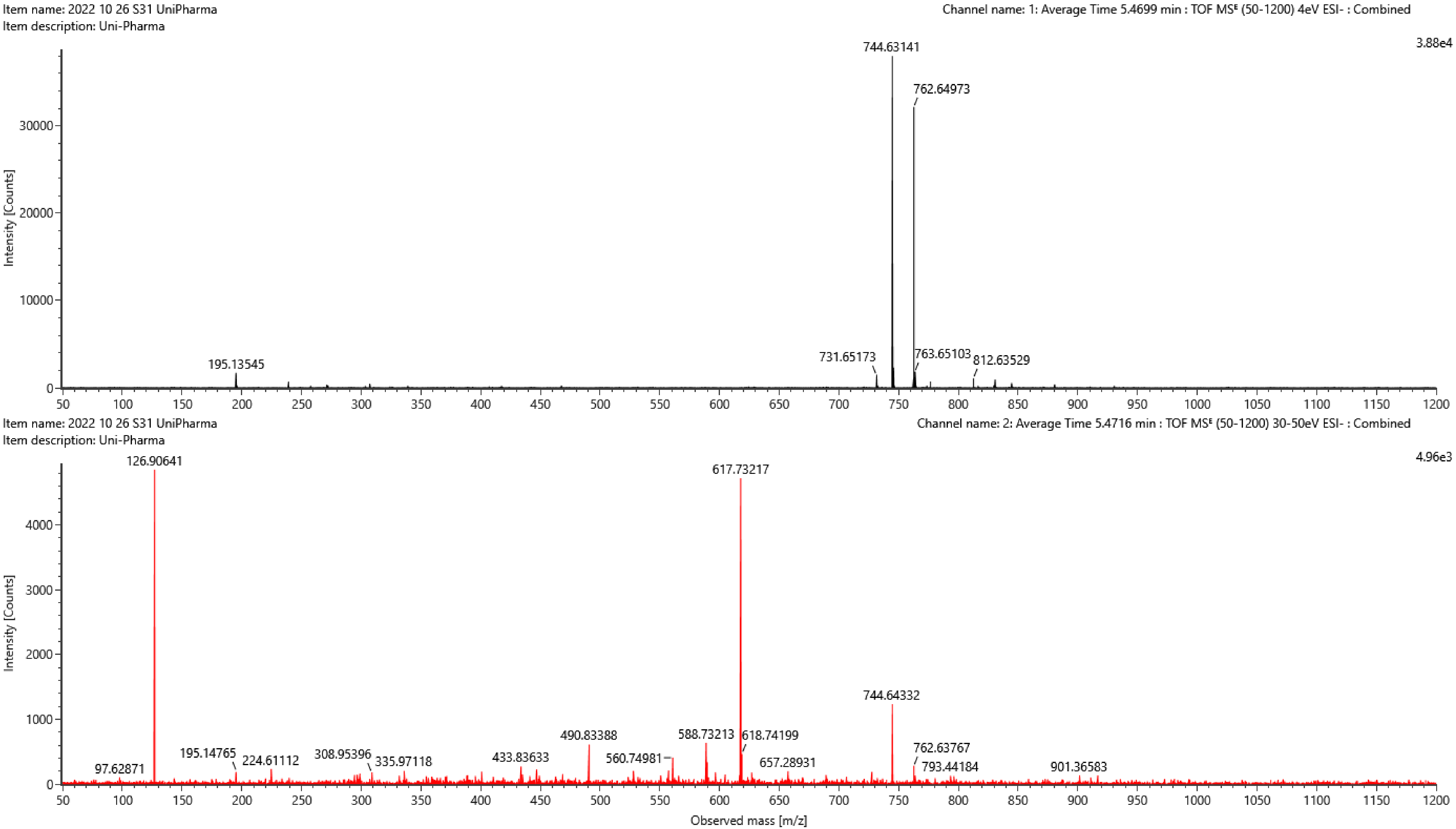

Figure S21: MS and MS/MS spectra of 2-Hydroxy-2-(4-(4-hydroxy-3,5-diiodophenoxy)-3,5-diiodophenyl) acetic acid, ESI$^-$

Table S13: Mass spectral information to levothyroxine impurity No. 4.

**No. 4: 2-(4-(4-hydroxy-3,5-diiodophenoxy)-3,5-diiodophenyl) acetamide**			
**Molecular formula: $C_{14}H_9I_4NO_3$**	**estimated structure:** 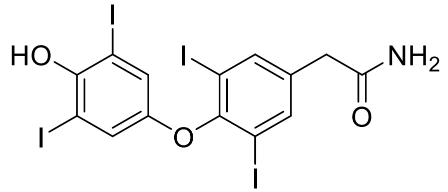		
**Retention time (min): 5.38**			
**Negative ion mode**		**Positive ion mode**	
**Measured mass [M-H]$^-$ (parent mass in MS spectra)**	**Mass error (Δppm)**	**Measured mass [M+H]$^+$ (parent mass in MS spectra):**	**Mass error (Δppm)**
745.6690	0.27	747.6845	1.47
**fragment ions in MS/MS spectra:**	**neutral loss:**	**fragment ions in MS/MS spectra:**	**neutral loss:**
618.7654<br>**574.7503***	I<br>**I + $CONH_2$**	**576.7669**	**I + $CONH_2$**

*bold = the most abundant fragment

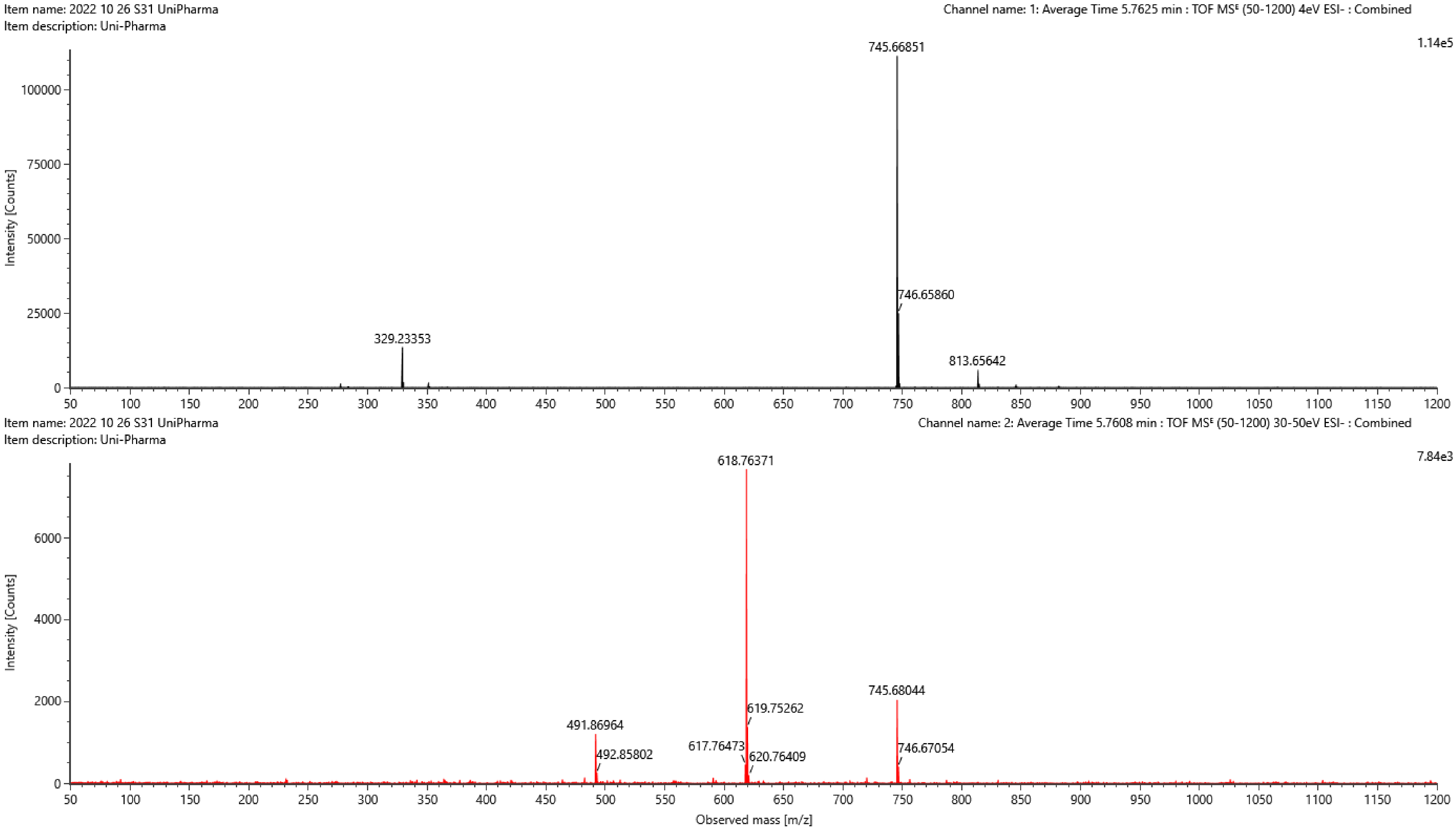

Figure S22: MS and MS/MS spectra of 2-(4-(4-hydroxy-3,5-diiodophenoxy)-3,5-diiodophenyl) acetamide, ESI^-

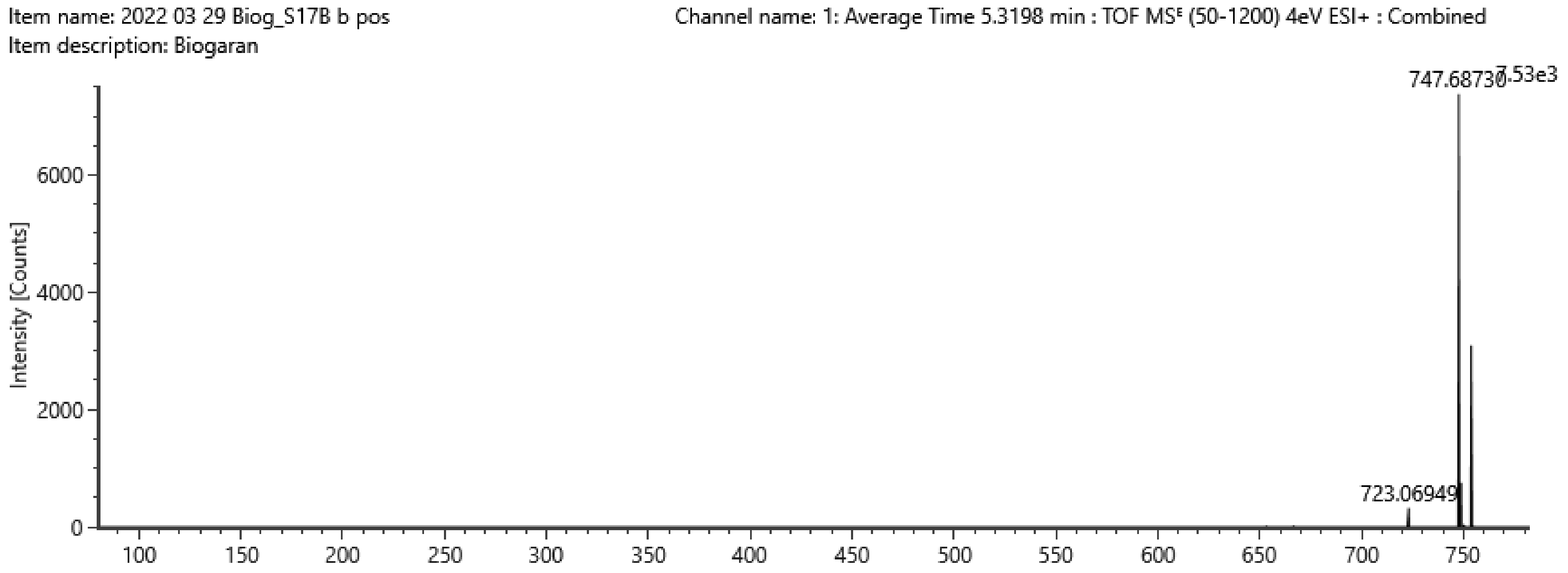

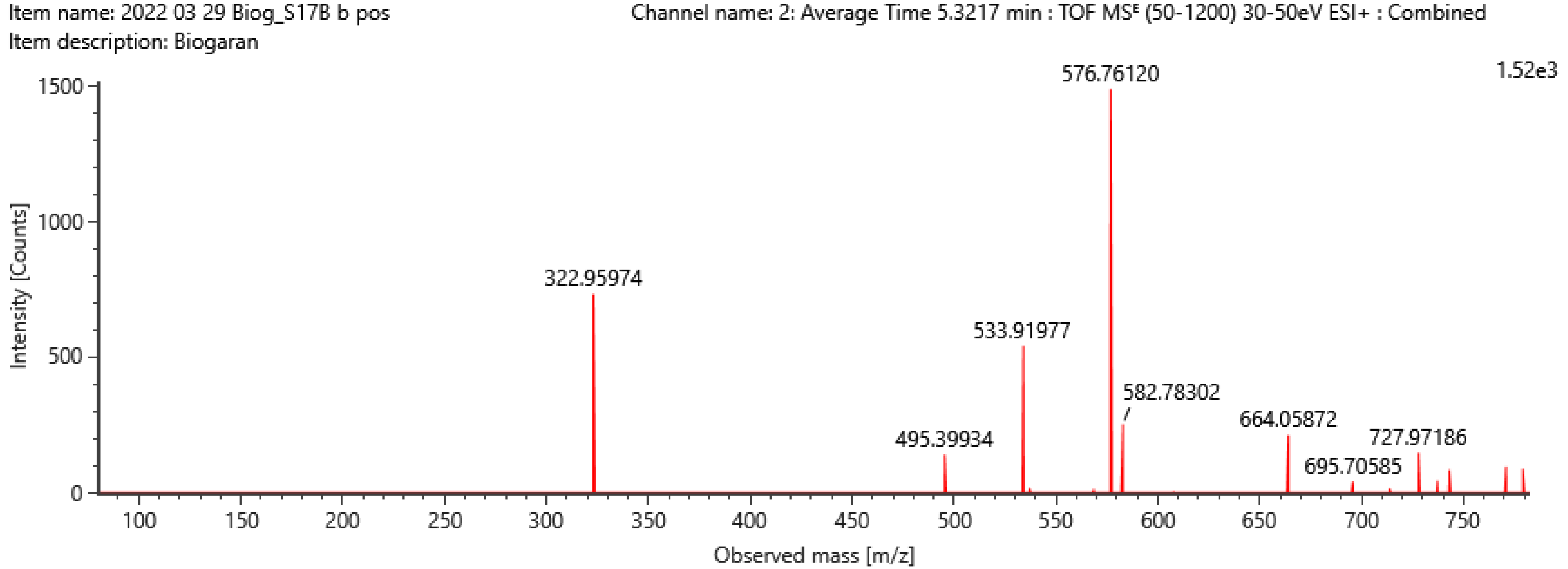

Figure S23: MS and MS/MS spectra of 2-(4-(4-hydroxy-3,5-diiodophenoxy)-3,5-diiodophenyl) acetamide, ESI^+

Table S14: Mass spectral information to levothyroxine impurity No. 5.

**No. 5: 2-(4-(4-hydroxy-3,5-diiodophenoxy)-3,5-diiodophenyl)-2-oxoacetamide**			
**Molecular formula: $C_{14}H_7I_4NO_4$**	**estimated structure:** 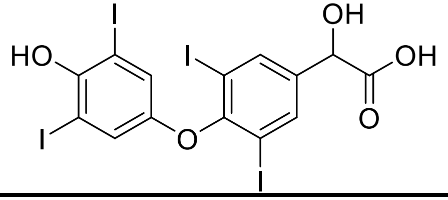		
**Retention time (min): 5.70**			
**Negative ion mode**		**Positive ion mode**	
**Measured mass [M-H]⁻ (parent mass in MS spectra)**	**Mass error (Δppm)**	**Measured mass [M+H]⁺ (parent mass in MS spectra):**	**Mass error (Δppm)**
759.6487	0.77	n.d.	-
**fragment ions in MS/MS spectra:**	**neutral loss:**	**fragment ions in MS/MS spectra:**	**neutral loss:**
**632.7450***<br>573.7444<br>561.7456	**I**<br>I + $CHNO_2$<br>I + C_2HNO_2	-	-

*bold = the most abundant fragment

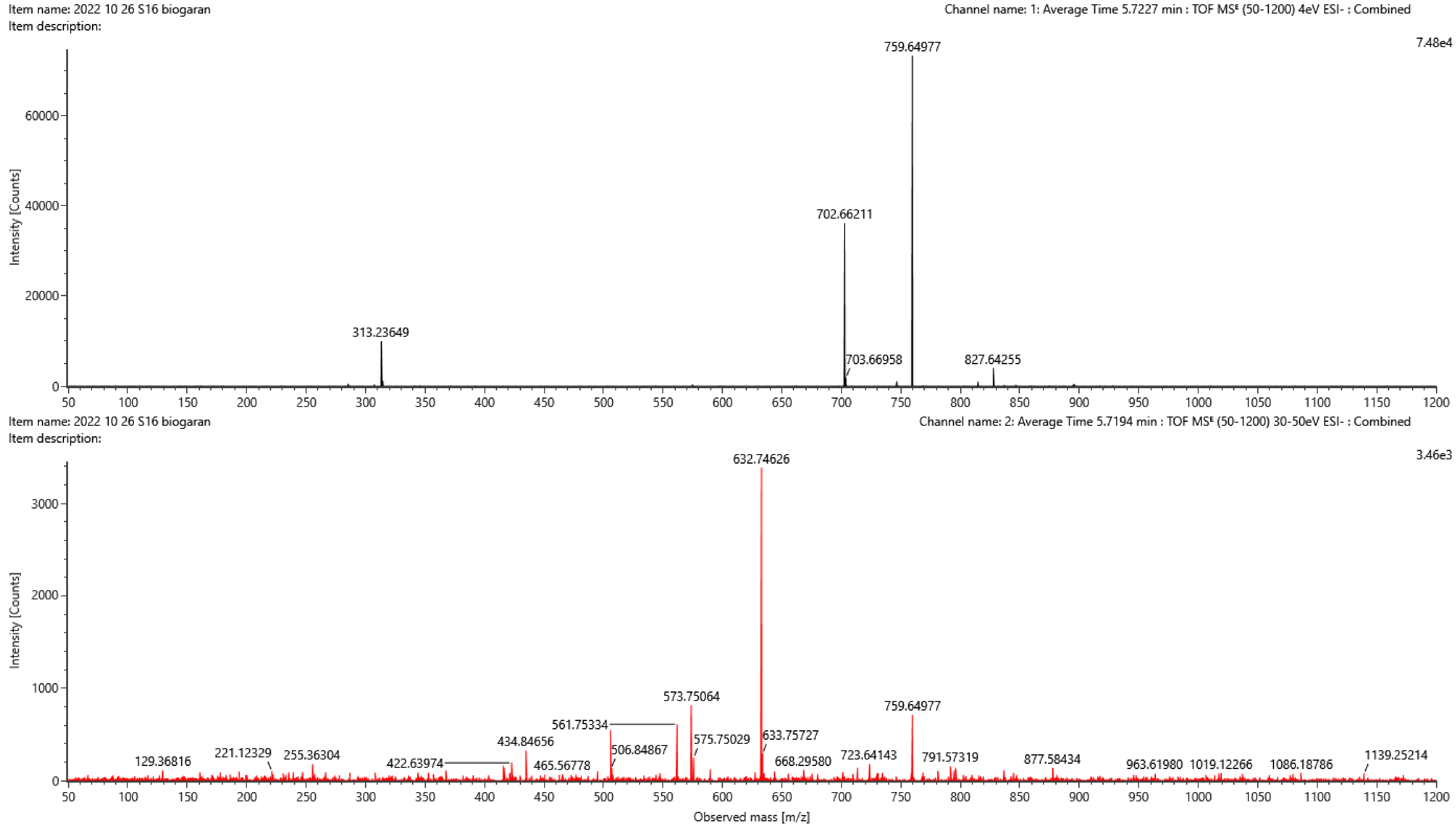

Figure S24: MS and MS/MS spectra of 2-(4-(4-hydroxy-3,5-diiodophenoxy)-3,5-diiodophenyl)-2-oxoacetamide, ESI$^-$

Table S15: Mass spectral information to levothyroxine impurity No. 6.

**No. 6: 4-(4-hydroxy-3,5-diiodophenoxy)-3,5-diiodobenzoic acid (EP impurity H)**			
**Molecular formula: $C_{13}H_6I_4O_4$**	**estimated structure:** 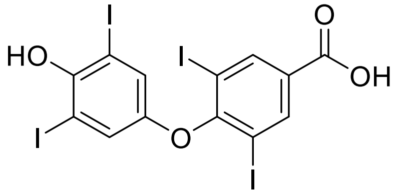		
**Retention time (min): 5.84**			
**Negative ion mode**		**Positive ion mode**	
**Measured mass $[M-H]^-$ (parent mass in MS spectra)**	**Mass error (Δppm)**	**Measured mass $[M+H]^+$ (parent mass in MS spectra):**	**Mass error (Δppm)**
732.6379	0.96	n.d.	-
**fragment ions in MS/MS spectra:**	**neutral loss:**	**fragment ions in MS/MS spectra:**	**neutral loss:**
688.6416<br>**605.7325***<br>560.7359<br>359.8152	CO_2<br>**I**<br>CO_2 + HI<br>$C_7H_4I_2O_2$	-	-

*bold = the most abundant fragment

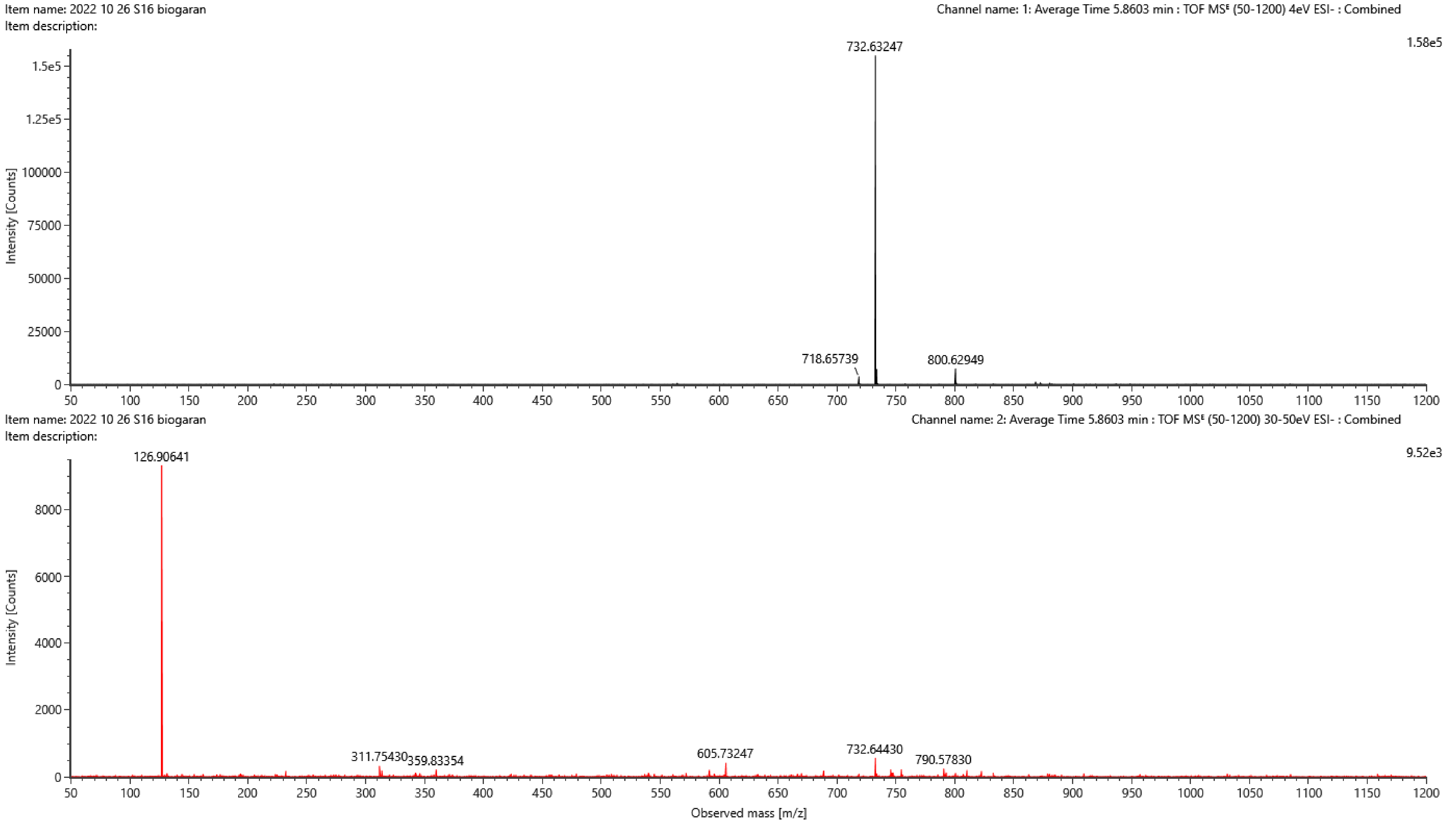

Figure S25: MS and MS/MS spectra of 4-(4-hydroxy-3,5-diiodophenoxy)-3,5-diiodobenzoic acid (EP impurity H), ESI$^-$

Table S16: Mass spectral information to levothyroxine impurity No. 7.

**No. 7: 4-(4-hydroxy-3,5-diiodophenoxy)-3,5-diiodo acetaldehyde**			
**Molecular formula: $C_{14}H_8I_4O_3$**	**estimated structure:** 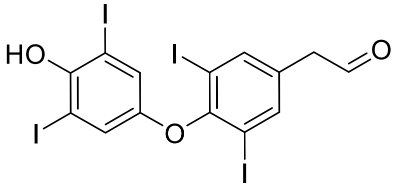		
**Retention time (min): 6.13**			
**Negative ion mode**		**Positive ion mode**	
**Measured mass [M-H]⁻ (parent mass in MS spectra)**	**Mass error (Δppm)**	**Measured mass [M+H]⁺ (parent mass in MS spectra):**	**Mass error (Δppm)**
730.6585	0.75	n.d.	-
**fragment ions in MS/MS spectra:**	**neutral loss:**	**fragment ions in MS/MS spectra:**	**neutral loss:**
603.7535<br>**574.7515***	I<br>**I + CHO**	-	-

*bold = the most abundant fragment

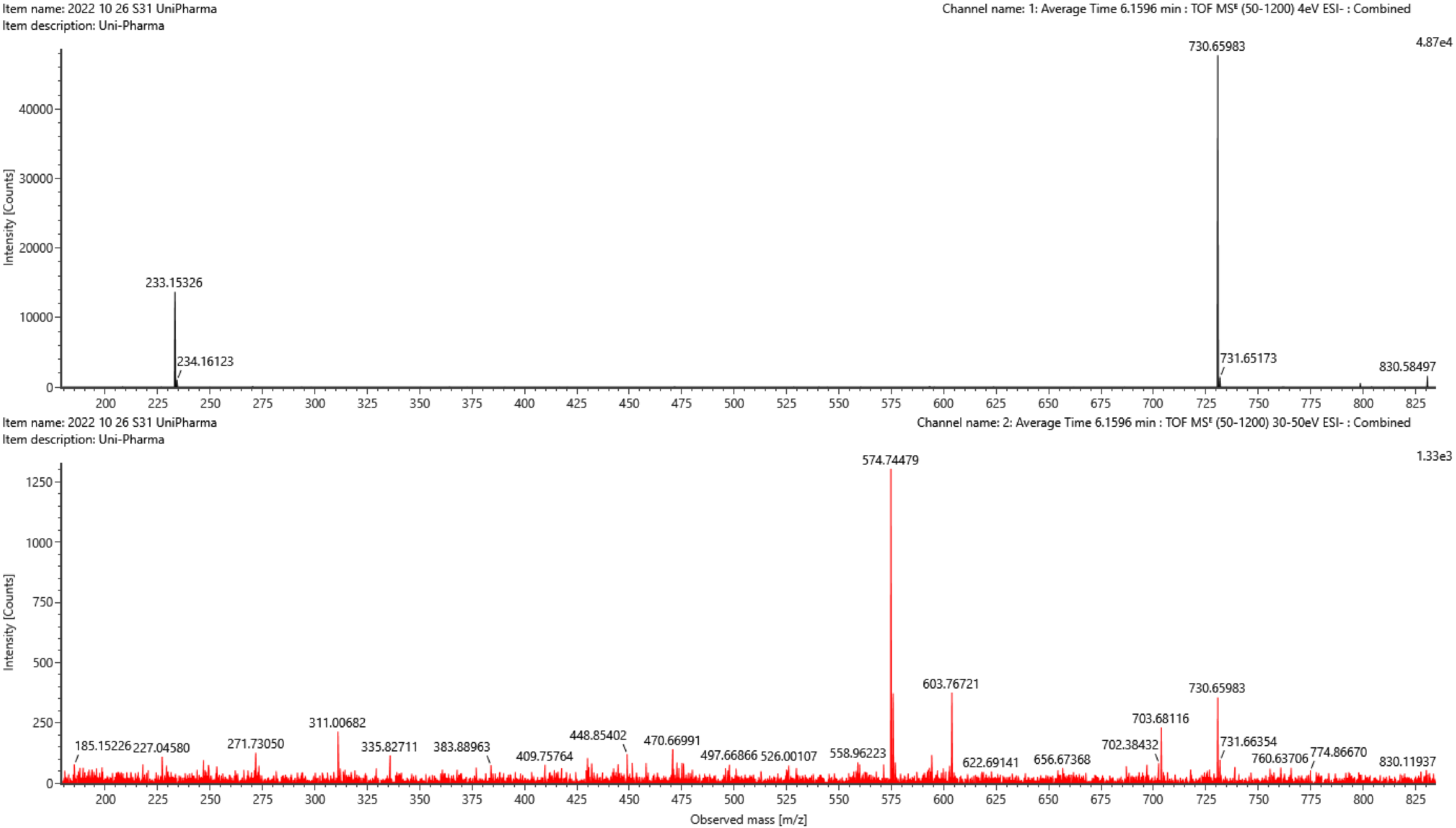

Figure S26: MS and MS/MS spectra of 4-(4-hydroxy-3,5-diiodophenoxy)-3,5-diiodo acetaldehyde, ESI⁻

Table S17: Mass spectral information to levothyroxine impurity No. 8.

**No. 8: 4-(4-hydroxy-3,5-diiodophenoxy)-3,5-diiodo benzaldehyde (EP impurity I)**			
**Molecular formula:** $C_{13}H_6I_4O_3$	**estimated structure:** 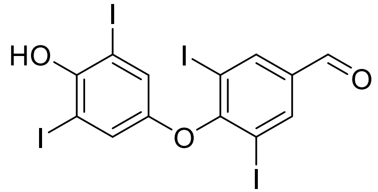		
**Retention time (min): 6.35**			
**Negative ion mode**		**Positive ion mode**	
**Measured mass [M-H]$^-$ (parent mass in MS spectra)**	**Mass error (Δppm)**	**Measured mass [M+H]$^+$ (parent mass in MS spectra):**	**Mass error (Δppm)**
716.6421	-0.28	n.d.	-
**fragment ions in MS/MS spectra:**	**neutral loss:**	**fragment ions in MS/MS spectra:**	**neutral loss:**
689.6626<br>**589.7340***<br>462.8332<br>372.8223	CHO<br>**I**<br>2I<br>$C_6H_2I_2O$	-	-

*bold = the most abundant fragment

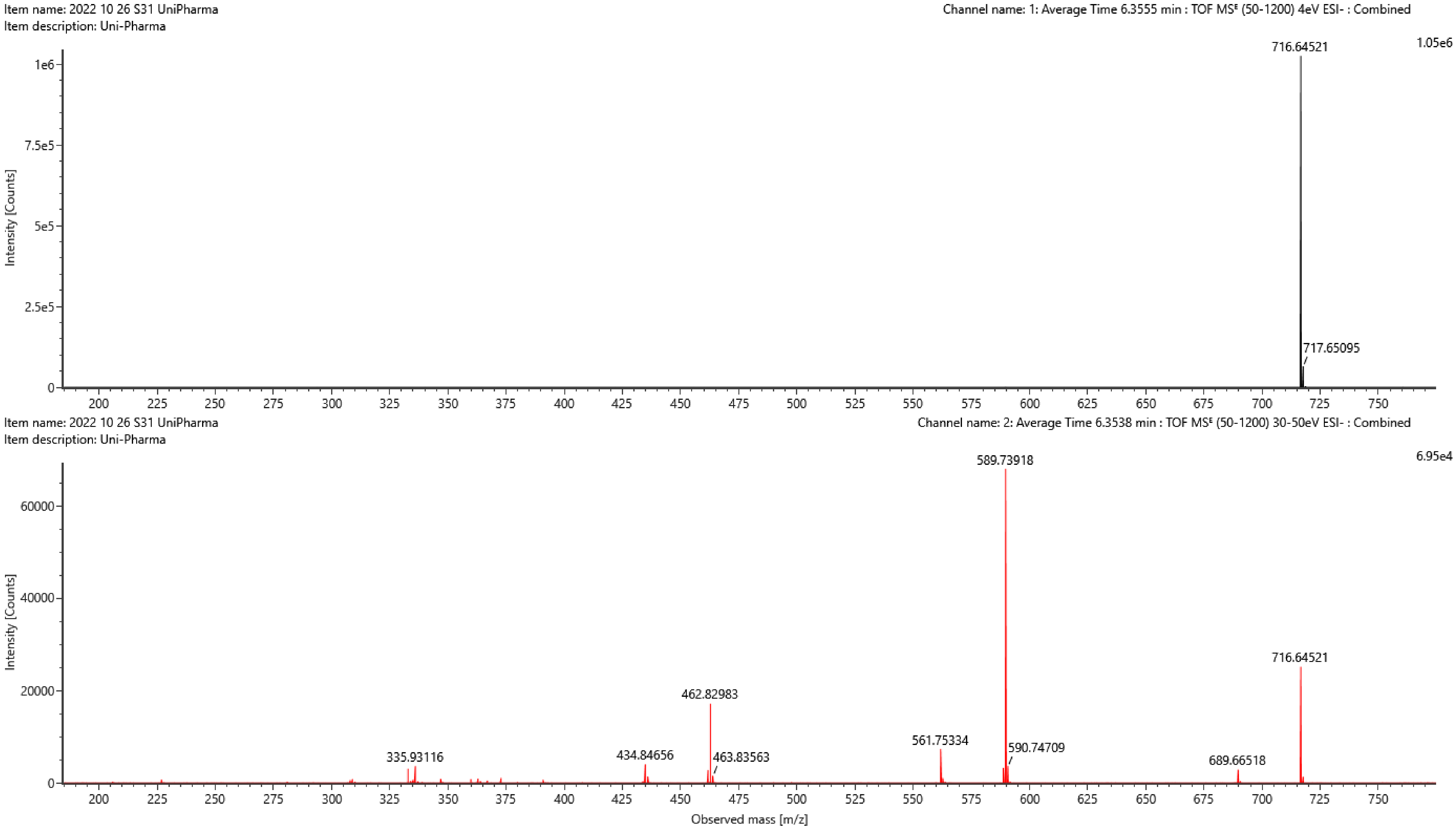

Figure S27: MS and MS/MS spectra of 4-(4-hydroxy-3,5-diiodophenoxy)-3,5-diiodo benzaldehyde (EP impurity I), ESI$^-$

Table S18: overview of relative intensities of levothyroxine-derived impurities, calculated based on MS response (the values above 1% are highlighted in bold) relative to API.

number	1	2	3	4	5	6	7	8
**m/z**	**1099.786**	**649.783**	**762.648**	**745.669**	**759.648**	**732.638**	**730.659**	**716.642**
**RT (min)**	**4.05**	**4.10**	**5.39**	**5.56**	**5.70**	**5.84**	**6.13**	**6.35**
**M1L**	**1.23%**	0.13%	0.09%	n.d.	0.13%	0.08%	0.17%	**6.38%**
**M1M**	n.d.*	0.12%	0.02%	n.d.	0.02%	n.d.	0.02%	**3.91%**
**M2**	n.d.	n.d.	0.46%	0.21%	**1.04%**	**2.32%**	0.33%	**28.99%**
**M3**	n.d.	n.d.	n.d.	n.d.	n.d.	n.d.	n.d.	**1.67%**
**M4**	n.d.	0.07%	0.46%	0.01%	0.06%	0.15%	0.13%	**6.34%**
